# Cerebellar structure and function abnormalities in 16p11.2 microduplication mice

**DOI:** 10.1093/braincomms/fcag156

**Published:** 2026-05-08

**Authors:** Cessily Hayes, Hunter Halverson, Krisha Keeran, Krislen Tison, Kamilla Jacobo, Asriya Karki, Isaias Herring, Sri Naga Swetha Tunuguntla, Martha Pace, Binh Doan, Hsiang Wen, Annette Klomp, Marisol Lauffer, Marie E Gaine, Krystal L Parker, Aislinn J Williams

**Affiliations:** Iowa Neuroscience Institute, University of Iowa, Iowa City, IA 52242, USA; Department of Psychiatry, Carver College of Medicine, University of Iowa, Iowa City, IA 52242, USA; Pharmaceutical Sciences and Experimental Therapeutics, College of Pharmacy, University of Iowa, Iowa City, IA 52242, USA; Iowa Neuroscience Institute, University of Iowa, Iowa City, IA 52242, USA; Department of Psychiatry, Carver College of Medicine, University of Iowa, Iowa City, IA 52242, USA; Iowa Neuroscience Institute, University of Iowa, Iowa City, IA 52242, USA; Iowa Neuroscience Institute, University of Iowa, Iowa City, IA 52242, USA; Department of Psychiatry, Carver College of Medicine, University of Iowa, Iowa City, IA 52242, USA; Iowa Neuroscience Institute, University of Iowa, Iowa City, IA 52242, USA; Department of Psychiatry, Carver College of Medicine, University of Iowa, Iowa City, IA 52242, USA; Neuroscience and Behavior, Rutgers University-Newark, Newark, NJ 07103, USA; Iowa Neuroscience Institute, University of Iowa, Iowa City, IA 52242, USA; Department of Psychiatry, Carver College of Medicine, University of Iowa, Iowa City, IA 52242, USA; Iowa Neuroscience Institute, University of Iowa, Iowa City, IA 52242, USA; Department of Psychiatry, Carver College of Medicine, University of Iowa, Iowa City, IA 52242, USA; Department of Biology, Upper Iowa University, Fayette, IA 52412, USA; Pharmaceutical Sciences and Experimental Therapeutics, College of Pharmacy, University of Iowa, Iowa City, IA 52242, USA; Iowa Neuroscience Institute, University of Iowa, Iowa City, IA 52242, USA; Department of Psychiatry, Carver College of Medicine, University of Iowa, Iowa City, IA 52242, USA; Iowa Neuroscience Institute, University of Iowa, Iowa City, IA 52242, USA; Department of Psychiatry, Carver College of Medicine, University of Iowa, Iowa City, IA 52242, USA; Iowa Neuroscience Institute, University of Iowa, Iowa City, IA 52242, USA; Iowa Neuroscience Institute, University of Iowa, Iowa City, IA 52242, USA; Department of Psychiatry, Carver College of Medicine, University of Iowa, Iowa City, IA 52242, USA; Pharmaceutical Sciences and Experimental Therapeutics, College of Pharmacy, University of Iowa, Iowa City, IA 52242, USA; Iowa Neuroscience Institute, University of Iowa, Iowa City, IA 52242, USA; Department of Psychiatry, Carver College of Medicine, University of Iowa, Iowa City, IA 52242, USA; Iowa Neuroscience Institute, University of Iowa, Iowa City, IA 52242, USA; Department of Psychiatry, Carver College of Medicine, University of Iowa, Iowa City, IA 52242, USA

**Keywords:** cerebellum, Purkinje cells, eyeblink conditioning, stellate cells, cerebellar learning

## Abstract

16p11.2 microduplication (16p11.2^dp/+^) is associated with neuropsychiatric disorders including schizophrenia, autism and intellectual disability. Cerebellar abnormalities have been implicated in these disorders. In 16p11.2^dp/+^ mice, the cerebellum displays significant transcriptional dysregulation, and humans with 16p11.2 microduplication have decreased cerebellar volume. Despite this, cerebellar anatomy and cerebellar-dependent behaviour in 16p11.2^dp/+^ mice remain uncharacterized. To address this, we histologically examined the cerebellar cortex in 16p11.2^dp/+^ mice. There were no structural differences in cerebellar lobule IV/V or impairments in gait or motor coordination, commonly associated with lobule IV/V. In contrast, more Purkinje cells (PCs) were mislocalized to the granule layer and parvalbumin expression was decreased in molecular layer interneurons (MLIs) in cerebellar lobule VI of 16p11.2^dp/+^ mice, but not in lobule IV/V. Cerebellar lobule VI is associated with delay eyeblink conditioning, and 16p11.2^dp/+^ mice are impaired in cerebellum-dependent associative learning on this task. Specifically, 16p11.2^dp/+^ mice had conditioned response (CR) percentage and CR onset latency deficits, suggesting lobule-specific alterations to PC localization and MLI parvalbumin expression may impair learning and adaptive timing of cerebellar-driven CRs. Similarly, schizophrenia involves CR acquisition deficits in delay eyeblink conditioning. Further investigation of the cerebellum in 16p11.2^dp/+^ mice may provide insights into the pathogenesis of neuropsychiatric disorders linked to this copy number variant.

## Teaser

Hayes *et al*. report that mice with a 16p11.2 chromosomal microduplication, relevant for schizophrenia, show cerebellar lobule VI-specific ectopic Purkinje cells and reduced parvalbumin expression in molecular layer interneurons. These mice demonstrate impaired delay eyeblink conditioning, suggesting cerebellar dysfunction may contribute to neuropsychiatric symptoms associated with this genetic variant.

## Graphical Abstract

**Graphical Abstract:**
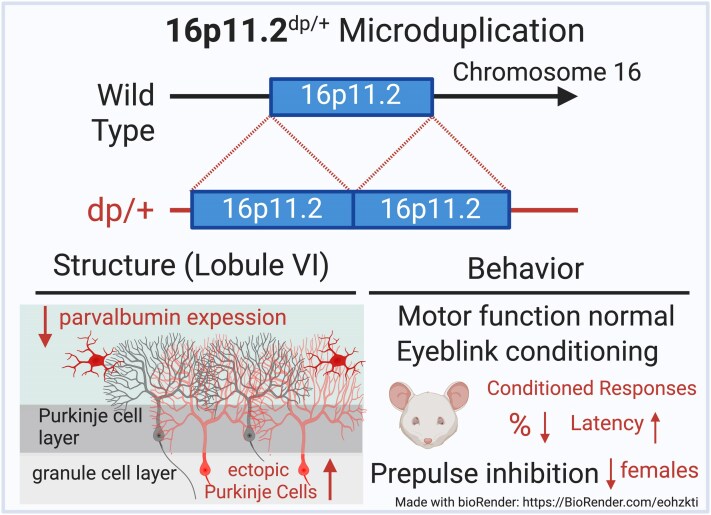

## Introduction

The World Health Organization estimates that one in eight people worldwide lives with a neuropsychiatric disorder (2019), yet the ontogenic mechanisms are poorly understood. Certain copy number variants (CNVs) increase the risk of developing one or multiple disorders and thus serve as a useful model to elucidate the shared aetiology of neuropsychiatric disorders. Although rare in the general population, with an estimated prevalence of 1 of every 4216 live births,^1^ 16p11.2 microduplication has been associated with multiple neurodevelopmental and neuropsychiatric disorders, including schizophrenia, autism spectrum disorder, bipolar disorder, attention deficit hyperactivity disorder (ADHD) and intellectual disability.^2^ Additionally, genome-wide association revealed 16p11.2^dp/+^ CNV confers high genetic risk for schizophrenia development.^3^

Frontal cortical abnormalities are well-established phenotypes of 16p11.2 microduplication^2,4-6^ and neuropsychiatric disorders,^7,8^ yet the precise cerebellar role is underexplored. While historically considered a primary regulator of motor control, evidence from cerebellar stroke, lesion and neuropsychiatric studies supports cerebellar involvement in non-motor functions. Abnormalities in posterior cerebellar lobules (lobules VI–X) contribute to dysfunction in executive function, linguistic processing and spatial cognition, while motor dysfunction arises from the anterior lobules (lobules I–IV/V).^9-16^

Humans and mouse model behavioural studies link the cerebellum with neuropsychiatric disorders relevant to 16p11.2 microduplication. Delay eyeblink conditioning (EBC) is the canonical test for cerebellar-dependent associative learning. Electrophysiological studies in animals show acquisition of conditioned responses (CRs) relies on long-term depression at the parallel fibre-Purkinje cell (PC) synapse and long-term potentiation at the mossy fibre-interpositus nucleus synapse,^17-19^ exemplifying the necessity of functional cerebellar circuitry for learning. Additionally, cerebellar lobule VI lesions impair learning and expression of CRs in both humans and animals.^20-23^ Therefore, EBC serves as a useful measure of cerebellar integrity in neuropsychiatric disorders. EBC studies in schizophrenia reveal alterations to cerebellar-dependent associative learning, including reduced CR %^24-28^ and longer CR onset latencies,^26,29^ whereas children with autism show shorter CR onset latencies.^30^ In animal models, the Erasmus Ladder provides a novel behavioural paradigm to evaluate cerebellar-dependent motor performance and learning within a single experiment. Cerebellar-dependent learning involves mice traversing a raised rung on the horizontal ladder, making a learned association between a tone and a perturbation. This requires the inferior olive^31^ and PCs,^32^ underscoring the crucial role of the cerebellum in this paradigm. Moreover, impairments in motor coordination and locomotor activity are associated with lesions in cerebellar lobule IV/V in mice.^33^ Mice lacking *Shank2* in PCs exhibit cerebellar-mediated learning deficits without changes in motor performance on Erasmus Ladder.^34^

Alterations in cerebellar structure are implicated in neuropsychiatric disorders, including those conferred by 16p11.2 microduplication. Post-mortem autism studies reveal reduced PC density and PC soma area in the cerebellar vermis and hemispheres.^35-37^ Post-mortem results are inconsistent in schizophrenia, some reporting PC abnormalities^38^ and others finding no loss of PCs.^39^ A few studies have shown smaller cerebellar size in humans with 16p11.2 microduplication.^40^ Furthermore, genes duplicated in the 16p11.2 locus are more highly expressed in the cerebellum versus other brain regions.^41^ The structural and functional involvement of the cerebellum in neuropsychiatric disorders associated with 16p11.2 microduplication is compelling, yet it lacks comprehensively defined molecular, cellular and functional significance.

Delineating the role of the cerebellum in this CNV can be achieved using animal models. Although the cerebellum was not a major focus, structural and behavioural phenotypes have been reported in the Horev *et al*. mouse model, including reduced size of basal forebrain, hypothalamus, medial septum and periaqueductal grey in 16p11.2^dp/+^ mice,^42^ mirroring microcephaly in individuals with 16p11.2 microduplication.^40^ Interestingly, transcriptomic data in 16p11.2^dp/+^ mice revealed the highest occurrence of differentially expressed genes in the cerebellum versus other brain regions.^43^ Behaviourally, 16p11.2^dp/+^ mice do not display motor coordination deficits,^44^ but do exhibit hypolocomotion, social and cognitive deficits and increased repetitive self-grooming.^6,42,44^ There are conflicting findings for prepulse inhibition (PPI) of the startle response.^6,44^ PPI involves a weak stimulus (prepulse) that suppresses the startle response to a subsequent stronger startle stimulus (pulse). This task assesses sensorimotor gating, the process of filtering irrelevant sensory information, and deficits in attenuation of the startle response are common in schizophrenia and autism.^45-47^ While some studies report no PPI impairments in 16p11.2^dp/+^ mice,^44^ others have reported PPI deficits specific to female 16p11.2^dp/+^ mice.^6^ Considering the strong association of schizophrenia with this CNV, and the PPI impairments reported in schizophrenia, the conflicting findings regarding PPI impairments in 16p11.2^dp/+^ mice are unexpected.

Given inconsistent findings for PPI and a clear, yet undefined, role for the cerebellum in the pathophysiology of neuropsychiatric disorders that involve 16p11.2 microduplication, there is an opportunity to elucidate the structure and function of the cerebellum in mice with this CNV. Here, we analyse cerebellar cellular composition, cerebellar-dependent motor function, associative learning and sensorimotor gating.

## Materials and methods

### Animals

Breeding pairs of 16p11.2^dp/+^ mice (Strain #:013129)^42^ were maintained on a C57BL/6J x129S1/SvImJ (referred to as B6129SF1/J) hybrid background (Jax Strain #:101043). To generate experimental animals, 16p11.2^dp/+^ mice were bred to wild-type (WT) mice to produce litters with both 16p11.2^dp/+^ and WT offspring. All mice were at least 10 weeks old at the time of testing. WT littermates were same-sex group housed under light cycle on/off at 0900/2100 DST (0800/2000 non-DST) with enriched paper bedding. Mice were provided with food and water ad libitum. Behavioural experiments were conducted during the animals’ light cycle. Separate mice were used for histology and behavioural experiments. Experiments were conducted according to the National Institute of Health guidelines for animal care and were approved by the Institutional Animal Care and Use Committee at the University of Iowa.

### Histology

Mice were anesthetized by intraperitoneal injections of 17.5 mg/mL ketamine/2.5 mg/mL xylazine at a dose of 0.1 mL per 20 g. Cardiac perfusions were performed with ice-cold 0.1 M phosphate buffer (PB; pH 7.4) followed by 4% paraformaldehyde (PFA) diluted in PB. Whole brains were dissected and postfixed in 4% PFA for 24 h, then cryoprotected in 30% sucrose diluted in PB for 72 h. Brains were rinsed with PB and frozen in optimal cutting temperature compound using dry ice in 2-methylbutane. Brains were stored at −80°C for at least 24 h before being serially sectioned sagittally through the cerebellar vermis at 20 μm, on a cryostat set at −20°C. We retained every fifth section; therefore, serial sections were approximately 100 μm apart. Sections were air dried and stored at −20°C until use.

### Immunohistochemistry

Four sections from each mouse were stained using primary antibodies: calbindin D28K anti-rabbit recombinant polyclonal (1:200; Thermo Fisher) and parvalbumin anti-mouse monoclonal (1:200; Swant). Neurogranin (1:200; Millipore) was used to label Golgi cells. Primary antibodies were diluted in blocking buffer (0.1% Triton-X in 5% normal donkey serum in PB) and incubated on slides in a humidified chamber at 4°C overnight. Sections were then labelled with secondary antibodies: donkey anti-rabbit 488 (1:500; Jackson), donkey anti-mouse 594 (1:500; Jackson) and DAPI (1:1000; Thermo Fisher). Secondary antibodies with DAPI were incubated at room temperature for 2 h in a humidified chamber. Sections were coverslipped with Prolong Diamond Antifade Mountant (Invitrogen). Analyses are defined in Supplementary Methods.

### Nissl staining

Two sections per mouse were stained with thionin. Sagittal cerebellar vermis sections were rehydrated in deionized (DI) H_2_O for 5 s. Slides were dipped in thionin stain for ∼90 s, rinsed in DI H_2_O, then in 70% alcohol for 5 min, 95% alcohol for 5 min, and 95% alcohol/acetic acid until achieving desired colour. Slides were rinsed twice in 100% alcohol for 5 min each and moved through three separate citrus clearing solvent baths for 5 min each before Permount coverslipping. Analyses are defined in Supplementary Methods.

### Behavioural procedures

To study behavioural phenotypes, we performed Erasmus Ladder (Noldus, Wageningen, The Netherlands), delay EBC, activity monitoring and PPI in 16p11.2^dp/+^ and WT mice. Please see Supplementary Methods for details.

### Statistics

Full descriptions of the statistical analyses used are in the Supplementary Methods. All analyses were completed in RStudio, SPSS or GraphPad Prism 10.6.

Data are graphically represented as mean ± standard deviation (SD) for each group. Results were considered significant when *P* < 0.05 (denoted as follows: *P < 0.05; **P < 0.01). Non-significant data and follow-up tests are included in Supplementary Methods. Supplementary Table 1 includes all histology statistics. Supplementary Table 2 contains all behavioural data statistics.

## Results

### 16p11.2^dp/+^ mice display an increase in ectopic Purkinje cells in the granule layer of lobule VI

**Figure 1:**
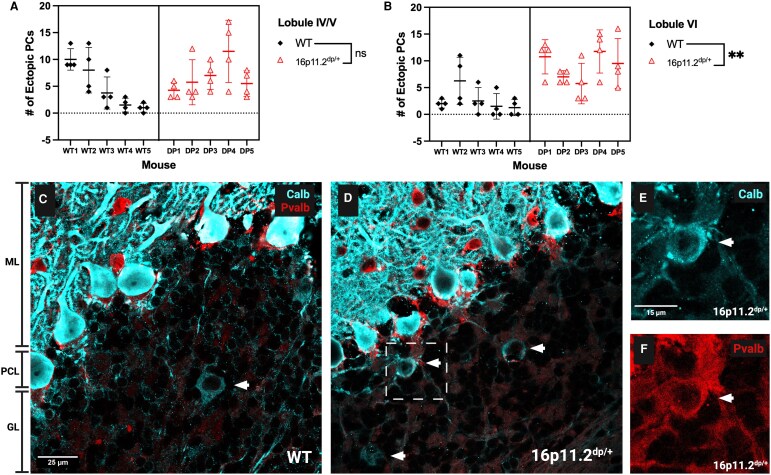
**Lobule VI-specific increase in calbindin+/parvalbumin+ ectopic PCs in 16p11.2^dp/+^ mice.** (**A**) No group differences were found in the number of ectopic PCs in lobule IV/V. (**B**) We observed a significant increase in ectopic PCs in the granule layer of lobule VI in 16p11.2^dp/+^ mice compared to WT littermates (*t*(8) = 4.308, *P* = 0.0026). (**A** and **B**) Nested *t*-test; mean ± SD, *n* = 10 (WTs: *n* = 3 females, *n* = 2 males; 16p11.2^dp/+^: *n* = 2 females, *n* = 3 males), age = 3.3–3.5 months. Each dot represents an individual measurement from each mouse. (**C**) Representative 40× confocal image of calbindin (calb), cyan, and parvalbumin (pvalb), red, immunostaining in lobule VI in WT tissue. White arrow indicates calbindin/parvalbumin+ ectopic PC in the granule layer. ML, molecular layer; PCL, Purkinje cell layer; GL, granule layer. (**D**) Representative 40× confocal image of calbindin and parvalbumin immunostaining of lobule VI in 16p11.2^dp/+^ mice. White arrows indicate calbindin/parvalbumin+ ectopic PCs in the granule layer. (**E** and **F**) Close up of ectopic PC in white box from (**D**) exhibiting both calbindin (**E**) and parvalbumin immunoreactivity (**F**). Created in BioRender. Williams lab, A. (2026) https://BioRender.com/xxd9mvh.

Structurally, cerebellar foliation and structure were normal in the cerebellum of 16p11.2^dp/+^ mice relative to WT, but ectopic calbindin+/parvalbumin+ PCs were found throughout the granule layer in both groups (Fig. 1). 16p11.2^dp/+^ mice had more calbindin+/parvalbumin+ ectopic PCs in the granule layer of cerebellar lobule VI (posterior cerebellum) (nested *t*-test, *P* = 0.0026; WT mean = 2.700, 16p11.2^dp/+^ mean = 8.950) but not in lobule IV/V (Fig. 1A and B; Supplementary Table 1). Ectopic PCs had inelaborate dendritic arbours (Fig. 1C and D) and appeared less intensely labelled for calbindin and parvalbumin than typically localized PCs (Fig. 1E and F). There were no differences in the density or size of typically localized PCs (Supplementary Fig. 1A and B and Table 1). Ectopic PCs in 16p11.2^dp/+^ mice were smaller than typically localized PCs (Supplementary Fig. 1C and Table 1). These cells could be detected using thionin but their contours were largely obscured by the surrounding granule cells (Supplementary Fig. 2A). These cells could be distinguished from Golgi cells, another large neuronal type in the granule layer, which express neurogranin (Supplementary Fig. 2B). Additional examples of ectopic PCs with poorly developed dendritic arbours are shown in Supplementary Fig. 2C.

### Lobule VI-specific decrease in apical parvalbumin+ molecular layer interneurons in 16p11.2^dp/+^ mice

**Figure 2:**
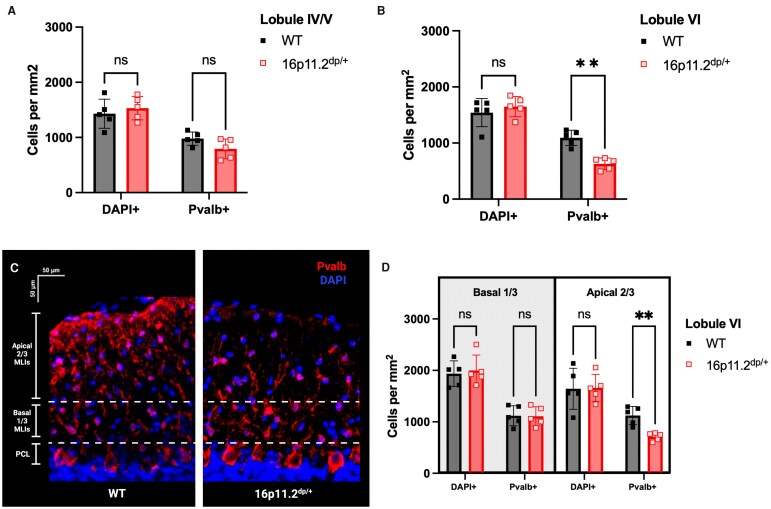
**Lobule VI-specific decrease in parvalbumin+ MLI cell counts, driven by apical MLIs, with no change in total molecular layer cell density in 16p11.2^dp/+^ mice.** (**A**) No group differences were found in DAPI+ nor parvalbumin+ (pvalb+) cell counts in lobule IV/V. (**B**) In lobule VI, parvalbumin+ (pvalb+) cell counts were significantly decreased in 16p11.2^dp/+^ mice compared to WT littermates (*t* ratio (8) = 5.9910, *P* = 0.0007), but DAPI+ cell counts were unchanged. (**C**) Representative 20× epifluorescent image of parvalbumin (pvalb), red, and nuclei (DAPI), blue, immunostaining of WT (left) and 16p11.2^dp/+^ (right) tissue in the molecular layer of lobule VI. Apical and basal molecular layer segments are indicated with dashed white lines and the PC layer is indicated as PCL. (**D**) In lobule VI, apical parvalbumin+ (pvalb+) cells were decreased in 16p11.2^dp/+^ mice compared to WT littermates (*t* ratio (8) = 4.795, *P* = 0.0054), but apical DAPI+, basal DAPI+, and basal parvalbumin+ cell counts were unchanged. (**A**, **B**, and **D**) Multiple unpaired *t*-tests, Holm-Sidak method; mean ± SD, *n* = 10 (WTs: *n* = 3 females, *n* = 2 males; 16p11.2^dp/+^: *n* = 2 females, *n* = 3 males), age = 3.3–3.5 months. Each dot represents the mean value from each mouse. Created in BioRender. Williams lab, A. (2026) https://BioRender.com/wutpmi2.

WT and 16p11.2^dp/+^ mice did not differ in DAPI+ cell counts or parvalbumin+ molecular layer interneuron (MLI) counts in lobule IV/V (Fig. 2A; Supplementary Table 1). However, in lobule VI, 16p11.2^dp/+^ mice had fewer parvalbumin+ MLIs than WT (multiple unpaired *t*-tests, Holm–Sidak method, parvalbumin+ cells: *P* = 0.0007) (Fig. 2B). There was no difference in total cell number in the molecular layer, as measured by DAPI, between 16p11.2^dp/+^ mice and WT in lobule VI (Fig. 2B; Supplementary Table 1).

Early born MLIs reside largely in the basal 1/3 of the molecular layer, while later-born MLIs mostly occupy the apical 2/3 of the molecular layer.^48^ When we divided the molecular layer into basal 1/3 versus apical 2/3 (Fig. 2C), we observed a significant reduction in parvalbumin+ apical MLI cells in 16p11.2^dp/+^ mice versus WT (multiple unpaired *t*-tests, Holm–Sidak method, *P* = 0.0054) (Fig. 2D). There were no differences in parvalbumin+ basal MLI cells or overall cell count in the basal 1/3 or apical 2/3 of the molecular layer (Fig. 2D; Supplementary Table 1).

### Cue-associated learning and motor learning impairments in 16p11.2^dp/+^ mice on Erasmus Ladder

**Figure 3:**
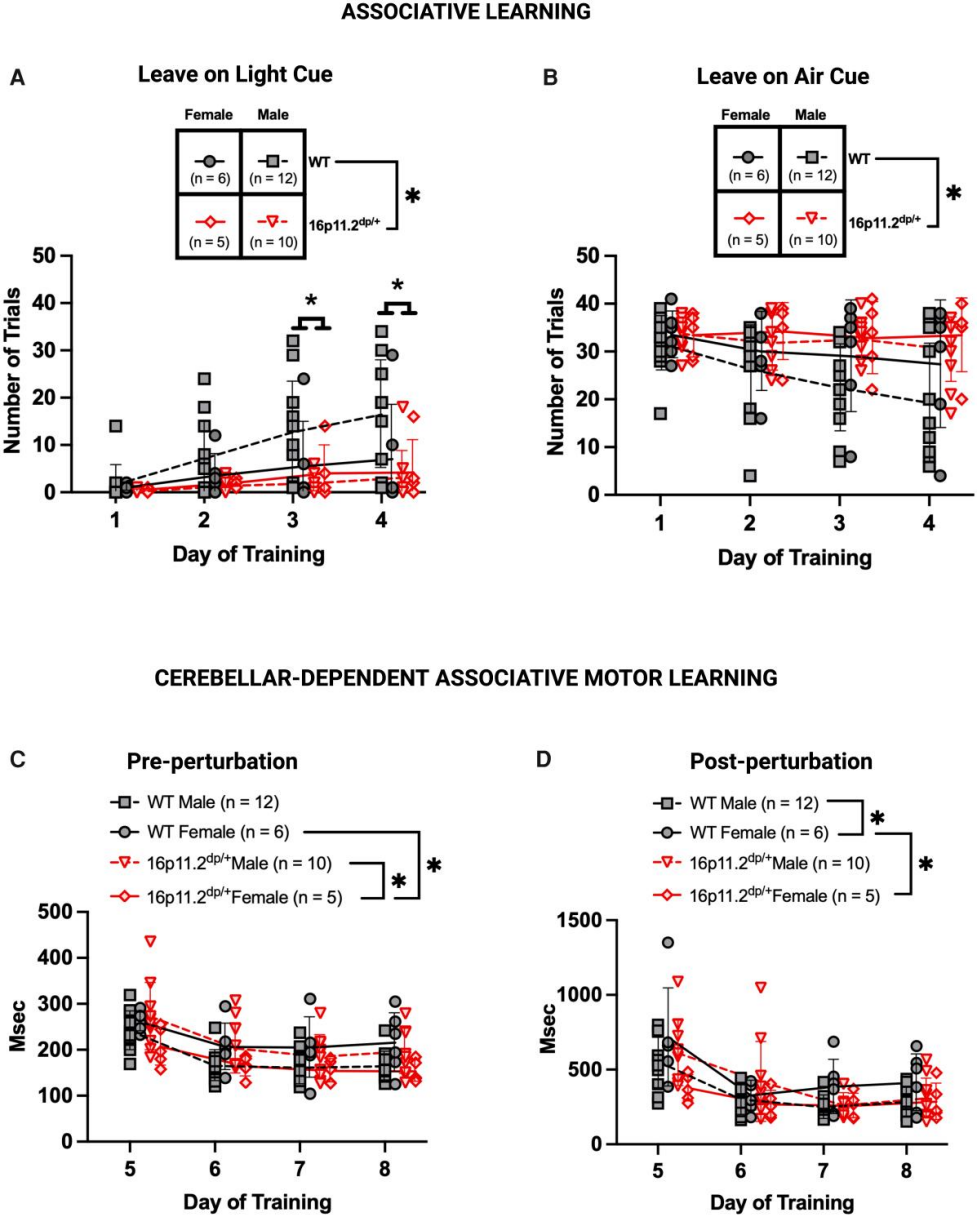
**Associative learning and cerebellar-dependent learning on Erasmus Ladder in 16p11.2^dp/+^ mice.** (**A**) WT mice left on light cue more frequently than 16p11.2^dp/+^ mice (*F*(1,29) = 4.98, *P* = 0.034). This difference appeared most prominent on Days 3 and 4 of training (asterisks) (*F*(3,86) = 14.35; Day 1 versus 3, *P* = 0.0001; Day 1 versus 4, *P* < 0.0001). (**B**) 16p11.2^dp/+^ mice left on air cue more frequently than WT littermates (*F*(1,29) = 4.64, *P* = 0.040). (**C**) Female 16p11.2^dp/+^ mice were significantly faster on the steps immediately preceding the perturbation than 16p11.2^dp/+^ males and WT females (*F*(1,28) = 8.49, female 16p11.2^dp/+^ versus male 16p11.2^dp/+^  *P* = 0.043, female 16p11.2^dp/+^ versus female WT *P* = 0.033). (**D**) Female 16p11.2^dp/+^ mice were significantly faster on the steps immediately following the perturbation than WT females, and WT females were significantly faster than WT males (*F*(1,28) = 7.69, female 16p11.2^dp/+^ versus female WT *P* = 0.015, female WT versus male WT *P* = 0.040). (**A–D**) Linear mixed effects modelling, Type III ANOVA Satterthwaite’s method. Mean ± SD, *n* = 33 (WTs: *n* = 6 females, *n* = 12 males; 16p11.2^dp/+^: *n* = 5 females, *n* = 10 males), age = 3.3–3.5 months. Created in BioRender. Williams lab, A. (2026) https://BioRender.com/r67rv3b.

The Erasmus Ladder assesses motor coordination, gait adaptation and cerebellar-dependent associative motor learning. It requires animals to traverse a ladder which assesses associative learning (responses to visual (light) and tactile (air puff) trial start cues) and motor functions (step times, gait adaptation, and tone-cued, cerebellar-dependent associative motor learning).^31^ For further details regarding equipment and experimental design, please see the Supplementary material. Although most animals initially did not respond to the light cue, they learned to use the light cue more frequently. WT mice learned to associate the light cue with trial start on Days 3 and 4 while 16p11.2^dp/+^ mice did not (Type III ANOVA Satterthwaite’s method, main effect of genotype, *F*_1,29_ = 4.9755, *P* = 0.0336; genotype by day interaction, *F*_3,86_ = 3.7700, *P* = 0.0136) (Fig. 3A; Supplementary Table 2). 16p11.2^dp/+^ mice left on the air cue more frequently than WTs (Type III ANOVA Satterthwaite’s method, main effect of genotype, *F*_1,29_ = 4.6359, *P* = 0.0398) (Fig. 3B). Therefore, WT mice learned to associate the light cue with the start of a trial more often, whereas 16p11.2^dp/+^ mice responded more frequently to the air cue. There were no differences between WT and 16p11.2^dp/+^ mice in missteps, step time or types of steps used (short versus long, also called gait adaptation) (Supplementary Fig. 3 and Table 2), although we did observe that female mice in general had faster step times than male mice (main effect of sex, *F*_(1,29)_ = 7.1035, *P* = 0.0125) (Supplementary Fig. 3B).

On Days 5–8 of the Erasmus Ladder, animals learn to jump over a tone-cued raised rung, termed the perturbation, which requires intact cerebellar function.^31^ For further details regarding equipment and experimental design, please see the Supplementary material. On Days 5–8, 16p11.2^dp/+^ females had faster step times than WT females and 16p11.2^dp/+^ males (Type III ANOVA Satterthwaite’s method, genotype by sex interaction, *F*_1,28_ = 8.4867, *P* = 0.0070) (Supplementary Table 2). All groups’ step times became significantly faster with training (Type III ANOVA with Satterthwaite’s method, main effect of day, *F*_3,84_ = 32.7951, *P* < 0.0001) (Fig. 3C). Post-perturbation, there was an interaction effect between genotype and sex driven by faster step times in both WT females versus WT males and WT females versus 16p11.2^dp/+^ females (Type III ANOVA Satterthwaite’s method, genotype by sex interaction, *F*_1,28_ = 7.6897, *P* = 0.0098) (Supplementary Table 2). All groups became significantly faster with training on Days 5–8 (Type III ANOVA with Satterthwaite’s method, main effect of day, *F*_3,84_ = 22.0529, *P* < 0.0001) (Fig. 3D). These data indicate tone-cued cerebellar motor learning occurred for all groups, but 16p11.2^dp/+^ females had faster step times than WT females and 16p11.2^dp/+^ males.

### Cerebellar-dependent associative learning impairments in 16p11.2^dp/+^ mice during eyeblink conditioning

**Figure 4:**
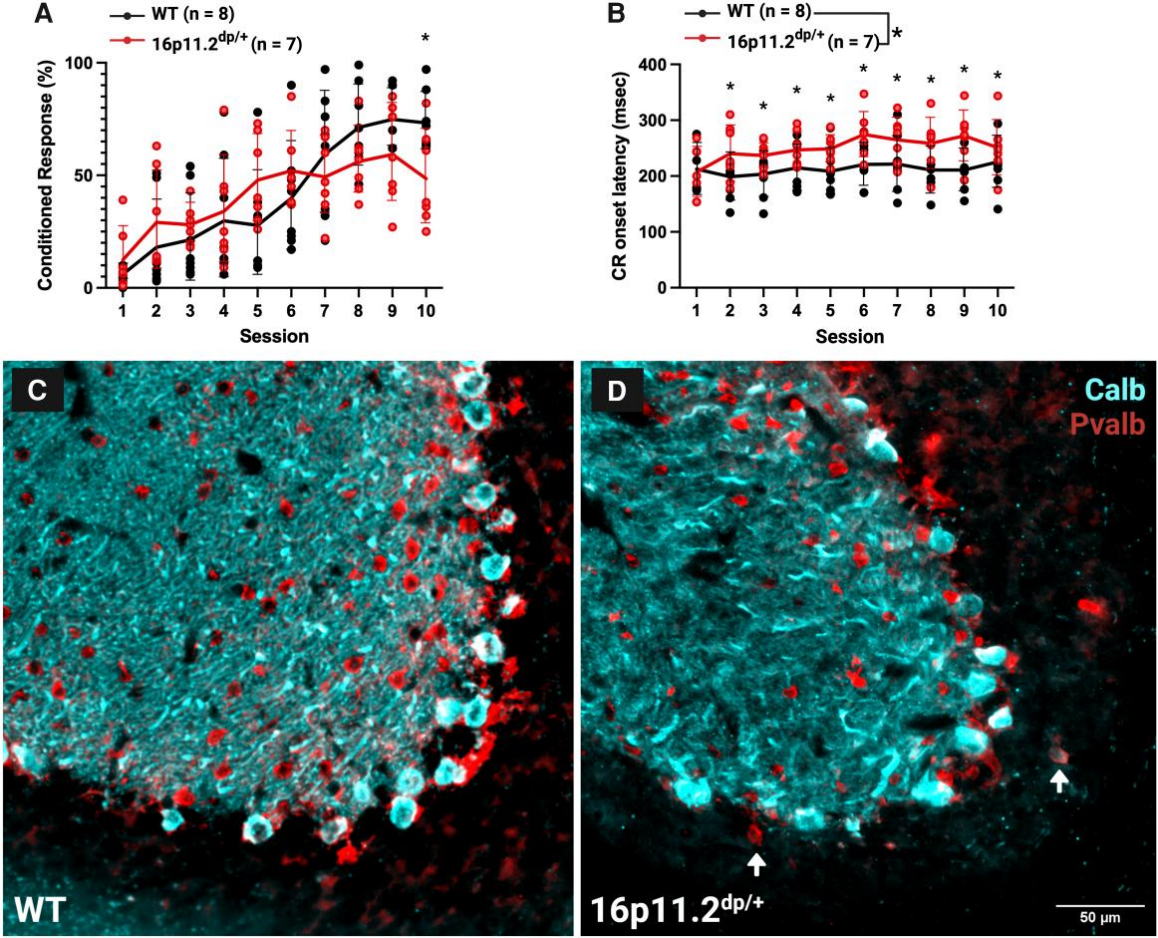
**Impaired CR acquisition and increased CR onset latencies during eyeblink conditioning in 16p11.2^dp/+^ mice.** (**A**) 16p11.2^dp/+^ mice showed fewer conditioned responses (CR) on Session 10 of training relative to WT mice (asterisk; Tukey’s multiple comparisons test, *P* = 0.023). (**B**) CR onset was longer in 16p11.2^dp/+^ mice than in WT littermates (main effect of genotype, *F*(1, 13) = 5.02, *P* = 0.043). (**A** and **B**) Data from both CS-US paired and CS alone trial types are shown. Mixed effects model (REML) with Geisser-Greenhouse correction, Type III fixed effects. Mean ± SD, *n* = 15 (WTs: *n* = 3 females, *n* = 8 males; 16p11.2^dp/+^: *n* = 5 females, *n* = 2 males), age = 4.8–6.8 months. (**C** and **D**) Representative 40× epifluorescent images of calbindin (calb), cyan, and parvalbumin (pvalb), red, immunostaining in the eyeblink microzone near the base of lobule VI in WT (**C**) and 16p11.2^dp/+^ (**D**) tissue. (**D**) White arrows indicate calbindin/parvalbumin+ ectopic PCs in the granule layer. Created in BioRender. Williams lab, A. (2026) https://BioRender.com/dj7y51b.

Cerebellar learning and timing were measured using delay EBC in 16p11.2^dp/+^ mice. Data were analysed using a mixed effects model (REML) with Geisser-Greenhouse correction, as sphericity was not assumed (ε = 0.2461). There was a significant main effect of session (*F*(2.215, 28.79) = 26.09, *P* < 0.001), indicating that both groups increased CR percentage across sessions, and no interaction between CR percentage and sex (Supplementary Table 2). There was no significant main effect of genotype. We observed a significant genotype by session interaction for CR percentage for EBC acquisition (*F*(2.215, 28.79) = 3.692, *P* < 0.034). Tukey’s multiple comparisons test revealed more CRs for WT than 16p11.2^dp/+^ mice on Session 10 [mean difference = 24.68, 95% CI (4.125, 45.23), *P* = 0.023], showing that 16p11.2^dp/+^ mice failed to reach the same level of learning as the WT by the final session (Fig. 4A). Therefore, 16p11.2^dp/+^ mice did not achieve the same level of cerebellar-dependent learning.

CR onset latency was analysed using a mixed effects model (REML) with Geisser-Greenhouse correction, as sphericity was not assumed (ε = 0.2208). There was no significant main effect of session (*F*(1.987, 25.83) = 2.508, *P* = 0.101) and no significant genotype by session interaction (*F*(1.987, 25.83) = 1.464, *P* = 0.250). However, there was a significant main effect of genotype (*F*(1, 13) = 5.024, *P* = 0.043), with 16p11.2^dp/+^ mice exhibiting longer CR onset latencies compared to WT were significantly later [predicted means: 250.1 ms versus 212.8 ms; mean difference = −37.29, 95% CI (−73.23, −1.349); Fig. 4B; Supplementary Table 2].

Overall, 16p11.2^dp/+^ mice had fewer CRs over the course of training, and onset latency was consistently increased, indicating impaired CR expression and learned adaptive timing during cerebellar-driven behaviour. Cerebellar control of delay EBC is linked to the eyeblink microzone, found near the base of lobule VI.^49^ Consistent with our previous observations (Figs 1B and 2B), the eyeblink microzone at the base of the primary fissure also shows ectopic PCs and decreased expression of parvalbumin in apical MLIs (Fig. 4C and D). We did not observe differences in mean eyeblink amplitude between groups or for CS alone trials (Supplementary Fig. 4).

### Mild hypoactivity in 16p11.2^dp/+^ mice during activity monitoring

Previous studies have reported decreased spontaneous activity in 16p11.2^dp/+^ mice.^42,50^ We performed home cage activity monitoring and observed mild reductions in spontaneous locomotor (horizontal) activity in 16p11.2^dp/+^ mice during the light phase but not the dark phase (Supplementary Fig. 5A and B and Table 2). We observed a genotype × hour interaction effect in average vertical (Z) activity hour-by-hour between WT and 16p11.2^dp/+^ littermates (genotype × hour interaction, *F*_(3.489,101.172)_ = 2.575, *P* = 0.049), and follow-up testing revealed a difference only at zeitgeber hour 10 (*F*_(1,29)_ = 5.468, *P* = 0.026) (Supplementary Fig. 5C and Table 2). We observed no difference in rearing (vertical) activity between groups when collapsing the data into light phase versus dark phase (Supplementary Fig. 5D and Supplementary Table 2).

### Impaired sensorimotor gating in female 16p11.2^dp/+^ mice during prepulse inhibition

**Figure 5:**
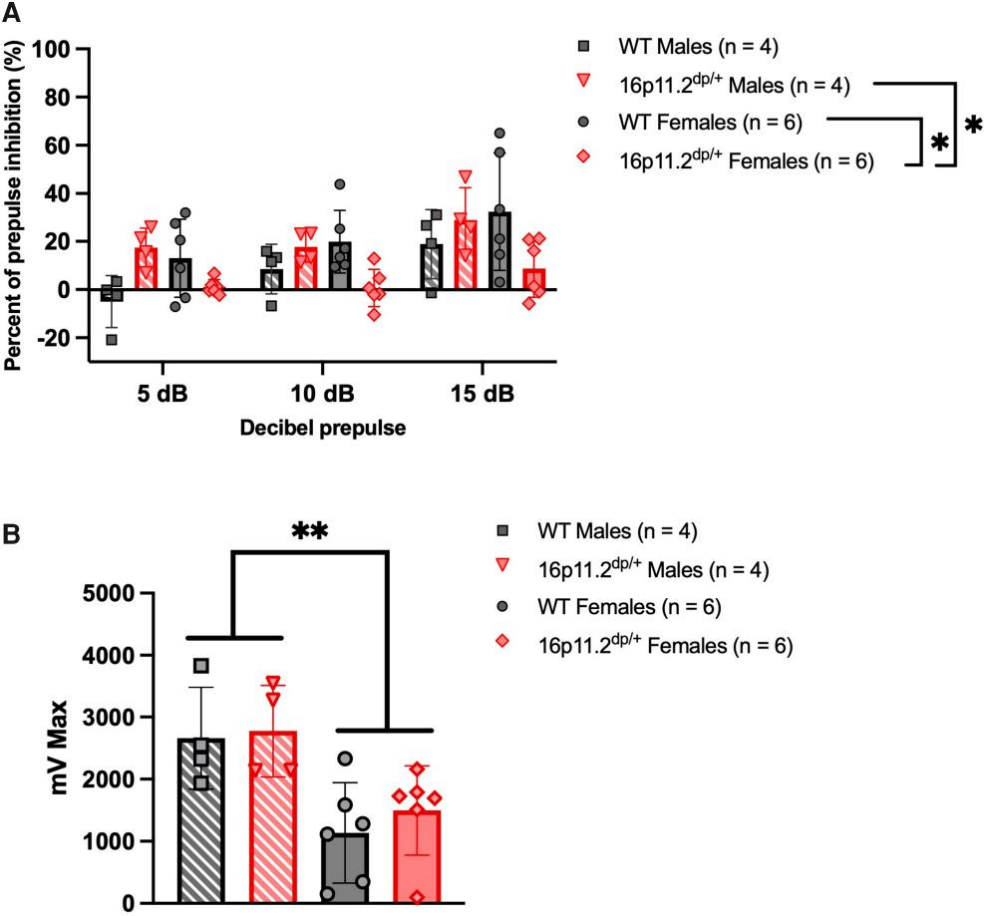
**Impaired sensorimotor gating with normal auditory startle in 16p11.2^dp/+^ mice.** (**A**) Female 16p11.2^dp/+^ showed reduced PPI relative to WT females and 16p11.2^dp/+^ males (*F*(2,32) = 3.30, female 16p11.2^dp/+^ versus female WT *P* = 0.014, female 16p11.2^dp/+^ versus male 16p11.2^dp/+^  *P* = 0.029). (**B**) Male mice display a greater startle response than female mice (block II shown) (*F*(1,16) = 12.64, *P* = 0.0026). (**A** and **B**) Linear mixed effects modelling, Type III ANOVA Satterthwaite’s method. Mean ± SD, *n* = 20 (WTs: *n* = 6 females, *n* = 4 males; 16p11.2^dp/+^: *n* = 6 females, *n* = 4 males), age = 2.7–4.8 months. Each dot represents the mean value from each mouse. Created in BioRender. Williams lab, A. (2026) https://BioRender.com/4e949xo.

16p11.2 microduplication is linked to neuropsychiatric disorders with reduced sensorimotor gating and impaired PPI including schizophrenia and autism.^45-47^ We observed an expected main effect of decibel, such that higher decibel prepulses elicited greater PPI (Satterthwaite’s method, main effect of decibel, *F*_2,32_ = 19.7922, *P* < 0.0001). PPI was not consistent at 5 dB in WTs but was at 10 and 15 dB. There were no main effects of genotype or sex but there was a decibel by genotype interaction effect (Type III ANOVA Satterthwaite’s method, decibel by genotype interaction effect, *F*_2,32_ = 3.2950, *P* = 0.0500; Supplementary Table 2). Female 16p11.2^dp/+^ mice display reduced PPI versus WT females and 16p11.2^dp/+^ males [Type III ANOVA Satterthwaite’s method, genotype by sex interaction effect, *F*_1,16_ = 9.3566, *P* = 0.0075; WT females versus 16p11.2^dp/+^ females (*P* = 0.0144), 16p11.2^dp/+^ females versus 16p11.2^dp/+^ males (*P* = 0.0289)] (Fig. 5A; Supplementary Table 2). Therefore, there are sex-specific deficits in sensorimotor gating in female 16p11.2^dp/+^ mice relative to WT females and 16p11.2^dp/+^ males. We also examined startle response alone and found that all groups habituated to the startle stimulus, but the startle response was detectable throughout the experiment (Type III ANOVA Satterthwaite’s method, main effect of block, *F*_2,32_ = 15.2470, *P* < 0.0001). Male mice had higher auditory startle responses than female mice (Type III ANOVA Satterthwaite’s method, main effect of sex, *F*_1,16_ = 12.2470, *P* = 0.0026), with no genotype or interaction effect (Fig. 5B).

## Discussion

Studies of 16p11.2^dp/+^ mice have largely focused on cortical abnormalities, despite growing evidence of cerebellar alterations contributing to neuropsychiatric phenotypes. Here, we histologically and behaviourally define the effects of this CNV in the mouse cerebellum. We identified altered cerebellar structure and cerebellar-dependent behaviour in 16p11.2^dp/+^ mice. We found that this CNV leads to alterations in PC localization and expression of parvalbumin in apical MLIs in lobule VI. Furthermore, our behavioural data suggest that the 16p11.2^dp/+^ CNV does not impair gait or motor coordination but does impair cerebellar-dependent learning as measured by EBC. Impairments in PPI and the associative learning aspects of the Erasmus Ladder task in 16p11.2^dp/+^ mice may reflect similar phenotypes to human patients with disorders associated with this CNV such as deficits in sensorimotor gating,^45-47^ associative learning^51^ and cerebellar-dependent associative learning.^24-29^

### Possible cellular mechanisms underlying delay eyeblink conditioning deficits in 16p11.2^dp/+^ mice

Lobule VI is associated with cerebellar-dependent EBC and is also where we observe structural alterations; these results suggest a mechanistic link between structural and behavioural alterations in 16p11.2^dp/+^ mice.^20,21^ We did not find differences in lobule IV/V structure or motor behaviours, such as motor coordination.^33^ We did not analyse hemispheric structure or cell counts, although the hemispheres are relevant for cerebellar learning and neuropsychiatric disorders.^16,52,53^ Future work analysing the hemispheres will be important for determining the extent of these changes in the cerebellar cortex.

The cerebellum controls adaptive timing of CR expression during EBC by decreasing simple spike activity of specific PCs that receive complex spikes elicited by the US.^17,19,20,54^ 16p11.2^dp/+^ mice exhibited longer eyeblink CR onset latencies and impaired CR acquisition, suggesting impaired learning-related PC activity which could result from lobule VI-specific structural alterations. While the role of ectopic PCs and reduced parvalbumin expression in apical MLIs in EBC is unknown, alterations to either or both cell types could cause an imbalanced integration of neuronal activity that may alter simple and complex PC spikes and output to cerebellar nuclei from typically located PCs.

PC axons project to deep cerebellar nuclei before establishing the PC monolayer during development^55^; therefore, ectopic PCs may signal to cerebellar nuclei despite their location in the granule layer. The combined output from a population of PCs that receive common climbing fibre inputs converge into the deep cerebellar nuclei, and together with local MLIs makes a functional unit of cerebellar cortex. MLIs decrease the regularity of PC simple spikes and the number of complex spikes by targeted reduction of apical MLI GABAergic neurotransmission.^48^ This was shown to occur in a study where EBC CRs were decreased following lobule VI-specific MLI DREADD inactivation (∼20%).^53^ Further work is needed to assess the electrophysiological activity of ectopic PCs, typically located PCs, and apical MLIs during EBC to better understand the link between the structural and behavioural alterations in 16p11.2^dp/+^ mice.

Potential alterations of the adaptively timed output from a cerebellar cortical functional unit due to the invasion of excessive ectopic PCs could result in abnormal plasticity mechanisms, compromised PC synchrony and ultimately abnormal behaviour. Compromised plasticity (LTP/LTD) mechanisms and decreased learning-related PC synchrony as a result of the increase in ectopic PCs and decrease in parvalbumin-expressing neurons in the typical stellate cell region of lobule VI may be sufficient to disrupt behaviour in 16p11.2^dp/+^ mice.

### Ectopic Purkinje cell phenotypes in mice

Ectopic PCs with preserved cerebellar foliation and typically located PC density and size have been reported in studies of other mouse models. Similar to 16p11.2^dp/+^ mice, p35 knockout (KO) mice display ectopic PCs in the granule layer with normal cerebellar foliation and typically located PC density, although the lobule localization of these ectopic PCs was unspecified.^56^ Other mouse models with ectopic PCs display reduced or no cerebellar foliation, ectopic PC clusters, lack of a PC monolayer, and aberrant lamina, including *Reeler*,^57-59^  *Scrambler*,^60,61^  *VLDLR/ApoE2*,^62^  *Src/Fyn*,^63^  *Staggerer*,^64^  *Pten*,^65^  *SmoA2*^66,67^ and *Cxcr4-/SDF-1* deficient mice.^68-70^ All these mutations disrupt PC migration via diverse mechanisms including altered Reelin signalling (*Reeler*, *Scrambler*, *VLDLR/ApoE2* and *Src/Fyn*), Sonic hedgehog signalling (*SmoA2* and *Staggerer*) or Bergmann glia gene expression (*Pten*). 16p11.2^dp/+^ mice exhibit fewer ectopic PCs than these mutant mice. Interestingly, the expression of *Dab1* (*Scrambler* mutant) and *RORα* (*Staggerer* mutant) were decreased by about 10–11% in the cerebellum of 16p11.2^dp/+^ mice,^43^ which may contribute to the ectopic cell phenotypes observed in this CNV. While ectopic PC phenotypes vary, shared developmental and migratory genes could play a role in the generation of ectopic PCs. Further work will be necessary to elucidate the contributions of individual genes such as *Dab1* and *RORα* to the 16p11.2^dp/+^ cerebellar phenotype.

### Origins of ectopic Purkinje cells

The morphogenetic mechanism by which some PCs become ectopic in the granule layer of both WT and 16p11.2^dp/+^ mice is unknown. Small numbers of ectopic PCs are present in normal rats and rabbits throughout the cerebellum, including the cerebellar nuclei and the molecular layer.^71^ Excess ectopic PCs were reported in the molecular layer and arbour vitae in phospholipase D2 KO mice, with a smaller number in their WT littermates but typical cerebellar size and foliation.^71,72^

We suggest that ectopic PCs may become trapped in the intermediate zone during migration from the ventricular zone to the nascent cerebellar cortex between E18-P7, ending their developmental trajectory in the granule layer.^73^ Some trapped PCs may degenerate and undergo apoptosis, while others continue differentiating,^66^ albeit to a lesser extent than their typically located counterparts.^71^ Ectopic PCs express calbindin and parvalbumin and therefore display some aspects of typical differentiation, but have smaller somata. Further analysis is needed to determine the full morphology, such as dendritic arborization, and circuit functions of these aberrant PCs.

It is unclear why ectopic PCs were increased in 16p11.2^dp/+^ mice in lobule VI but not IV/V, or how they influence behaviour. Excess ectopic PCs in 16p11.2^dp/+^ mice could be due to differential gene expression associated with this CNV, altering the PC progenitor pool, PC migration or PC maturation. Cortical neurons from 16p11.2^dp/+^ mice display abnormal development, hypothesized to involve accelerated GABAergic development driven by *Taok2* (a duplicated gene on the 16p11.2 locus), and subsequent over-activity of JNK, leading to premature closure of the critical period.^74^ Similarly, accelerated GABAergic PC development could contribute to excess ectopic PCs in 16p11.2^dp/+^ mice. Further investigation is required to explore ectopic PC developmental time points and location in other cerebellar lobules.

### Typically located Purkinje cells in 16p11.2^dp/+^ mice

In contrast to our findings, reduced PC density and PC soma area in the cerebellar vermis and hemispheres have been reported in post-mortem brains of individuals with autism^35-37^ and schizophrenia.^38^ Others have reported no loss of typically located PCs in individuals with schizophrenia.^39^ In cortical neurons derived from 16p11.2^dp/+^ patient induced pluripotent stem cells (iPSCs), reduced soma size and dendritic length was observed.^75^ Our results may not match previous work due to focus on different brain regions (cerebellum versus forebrain), different models (mouse versus human versus iPSCs) or differing aetiologies of autism versus schizophrenia versus 16p11.2 microduplication. Exploration of the cerebellum in this CNV is required to define potential alterations to typically located PC morphology, such as changes in dendritic arborization.

### Reduced parvalbumin expression without loss of neuronal density in 16p11.2^dp/+^ mice

16p11.2^dp/+^ mice display reduced parvalbumin expression in the apical MLIs of lobule VI without an overall loss of MLI density. RNA-sequencing supports this finding, having detected a ∼10% reduction in parvalbumin expression in 16p11.2^dp/+^ cerebellum.^43^ While mRNA expression does not necessarily reflect protein expression, these data do appear to support each other. Another group found elevated parvalbumin expression in the prefrontal cortex at P14 in 16p11.2^dp/+^ mice but decreased parvalbumin expression in adults; the authors theorized that parvalbumin expression may be downregulated to restore typical excitation/inhibition balance secondary to accelerated GABAergic neuronal development.^74^ Validation of the loss of parvalbumin protein expression in cerebellar lobule VI of 16p11.2^dp/+^ mice is needed. Moreover, it is necessary to quantify parvalbumin+ and DAPI+ cell counts in other lobules to determine whether the decrease in parvalbumin+ MLI cell counts is present in other cerebellar lobules. Recent work has identified single-molecule fluorescence in situ hybridization (smFISH) markers that are potentially capable of distinguishing between MLI subtypes.^76,77^ We used parvalbumin+/DAPI+ immunoreactivity and molecular layer location to identify MLIs and discern them based on birth order, though it would be interesting to assess these smFISH markers in 16p11.2^dp/+^ mice.

### Reduced parvalbumin expression in other brain regions in humans and mouse models related to neuropsychiatric disorders

Studies of schizophrenia rat models [methylazoxymethanol acetate (MAM) treatment], autism mouse models (*Shank*) and schizophrenia post-mortem human brains report reduced parvalbumin expression and/or parvalbumin+ cell counts without loss of parvalbumin+ neuron density. In *Shank* autism mouse models, both parvalbumin+ cell numbers and parvalbumin protein expression were reduced in somatosensory cortex and striatum, but total neuron density was unchanged.^78^ MAM-treated rats displayed a significantly decreased density of parvalbumin+ interneurons in medial prefrontal cortex and ventral subiculum of the hippocampus; however, total cell counts were not reported.^79^ In schizophrenia subjects, *in situ* hybridization and autoradiography revealed reduced parvalbumin mRNA expression but no change in total neuron number in prefrontal cortex. This reduction in parvalbumin mRNA expression was layer-specific, such that layer III and IV of the prefrontal cortex in schizophrenia exhibited significantly reduced parvalbumin mRNA expression while layers I, II and V/VI did not.^80^ Cortical layer development, similar to MLI development, proceeds in an inside-out fashion, such that later-born neurons migrate past early born.^81^ Parvalbumin+ cell density in the apical 2/3 of the molecular layer in lobule VI of the 16p11.2^dp/+^ mouse cerebellum is decreased, emulating the reduction of parvalbumin mRNA expression in later-born layers in other areas of the brain in individuals with schizophrenia.

### Functional cerebellar subregions and 16p11.2^dp/+^ mouse phenotypes

Lobule-specific phenotypes are found in functional subregions within the cerebellum. Mouse and human lesion studies, as well as fMRI studies in humans, point to functional gradients within the cerebellum, such that motor tasks are represented in anterior cerebellar lobules (lobule I—anterior lobule VI), with a second sensorimotor representation in lobule VIII, and non-motor tasks are represented in posterior lobules (lobule VI—Crus I; Crus II—VIIB; lobules IX-X).^82^

In humans, limb and gait ataxia are more often associated with stroke involving the superior cerebellar artery (supplying blood flow to anterior lobules) versus the posterior inferior cerebellar artery (supplying blood flow to posterior lobules).^9-11^ Additionally, anterior lobules, including anterior parts of lobule VI, along with lobule VIII, receive spinal afferents through the spinocerebellar tracts.^83^ These lobules are reciprocally interconnected with the motor cortices.^84^ Interestingly, individuals with infarction involving lobule VI and lobules VII-X, but not the anterior lobules, had a minor degree of motor impairment,^12^ suggesting lobule VI’s dual role in non-motor and, to a lesser extent, motor behaviours. Lesions of lobule IV/V in mice impair motor coordination and locomotor activity.^33^ In 16p11.2^dp/+^ mice, there were no gait or motor adaptation impairments on the Erasmus Ladder, and no alterations in ectopic PC number or parvalbumin+ MLI cell counts in lobule IV/V relative to WT.

Conversely, posterior inferior cerebellar artery stroke or damage to lobules VI, VII and IX results in cerebellar cognitive affective syndrome (CCAS), characterized by impairments in executive function, linguistic processing, spatial cognition and affect regulation.^13^ The CCAS model suggests the cerebellum maintains both motor *and* cognitive behaviour at a homeostatic baseline.^13^ This hypothesis applies to neuropsychiatric disorders associated with 16p11.2^dp/+^ CNV, such as schizophrenia, where alterations in the cortico-thalamic-cerebellar circuit are associated with cognitive dysfunction.^12,85,86^ Importantly, both behaviour and structure were altered in 16p11.2^dp/+^ mice in lobule VI. Further investigation of cognitive behaviours involving the cerebellum in 16p11.2^dp/+^ mice is crucial to link specific cerebellar structural abnormalities.

### Behavioural phenotypes in humans with neuropsychiatric disorders associated with 16p11.2 microduplication

Our data mirror some behavioural phenotypes in human neuropsychiatric disorders associated with 16p11.2 microduplication. Schizophrenia subjects show reduced CR%^24-28^ and longer CR latencies^26,29^ in EBC. Autistic children display typical trace EBC, but shorter latency CRs in delay EBC,^30^ contrasting our results showing longer latency CRs in 16p11.2^dp/+^ mice. CR indices tend to be lower in children with ADHD than controls.^87^ Schizophrenia^45,46^ and autism^47^ also involve reduced sensorimotor gating. Similarly, 16p11.2^dp/+^ mice exhibited PPI deficits but only in females. This finding contrasts with published reports showing reduced PPI% in males with schizophrenia and not females.^88^ Another group using the same 16p11.2^dp/+^ mouse model also found reduced PPI% predominantly in female 16p11.2^dp/+^ mice,^6^ while others report typical sensorimotor gating.^44^ The reasons for these discrepancies are unclear and could be secondary to differences in technical approach or housing conditions. Our data show that behavioural deficits in 16p11.2^dp/+^ mice resemble those found in humans with this CNV although the sex differences do not always align between species. We also found lobule-specific abnormalities in the cerebellar cortex, in both PCs and MLIs, that could contribute to these behavioural phenotypes. It will be important in future work to define the physiology of these cell types in 16p11.2^dp/+^ mice to fully understand how they are linked to behaviour. Our data suggest that 16p11.2^dp/+^ mice have face validity to test interventions for 16p11.2 microduplication-associated disorders, including forms of neuromodulation that could be translated to humans.

In conclusion, while there were no gross cerebellar structural alterations in 16p11.2^dp/+^ mice, we discovered lobule VI-specific differences in PC localization and reduced parvalbumin labelling in apical MLIs without a reduction in total molecular layer cells. Behaviourally, cerebellar-dependent EBC and female-specific sensorimotor gating in 16p11.2^dp/+^ mice were abnormal, with no alterations to gait or motor coordination. Together, our work supports cerebellar involvement in the pathogenesis of neuropsychiatric disorders associated with 16p11.2 microduplication.

## Supplementary Material

fcag156_Supplementary_Data

## Data Availability

The data that support the findings of this study are available from the corresponding author, upon reasonable request.
