# Supplementary material for: Cerebellar structure and function abnormalities in 16p11.2 microduplication mice
Source: Brain Commun. 2026 May 8;8(3):fcag156. doi: 10.1093/braincomms/fcag156 (PMC13175986; doi:10.1093/braincomms/fcag156)
Supplement: fcag156_Supplementary_Data [file fcag156_supplementary_data.pdf]

Supplementary Material

Methods

Histology

Ectopic Purkinje Cell Counts

Four immuno-labeled sagittal sections per mouse as described above were imaged at 20X in three locations (anterior, medial, and posterior) of lobule IV/V and lobule VI of the cerebellar vermis on an Olympus IX83 fluorescence microscope for the analysis of ectopic PCs. Twelve images per mouse (3 images/lobule/4 sections/mouse) were used. Counting was performed by raters blinded to genotype. Calbindin+ cells in the granule layer of cerebellar lobule IV/V and VI were classified as an ectopic PC if they met the following criteria: contained a nucleus, cell diameter between 10 – 20 μm, calbindin/parvalbumin immunoreactivity in the entire cell or the membrane, and the calbindin immunoreactivity of the ectopic PC must be distinct from background fluorescence. Ectopic PCs were counted manually in ImageJ for each image and recorded for both lobule IV/V and VI. Ectopic PC counts were added together within a section and resulted in a total of four data points per mouse.

Typically Located Purkinje Cell Cross-Sectional Soma Area

One thionin-stained sagittal section per mouse was imaged at 10X in lobule VI of the cerebellar vermis using bright field on an Olympus IX83 fluorescence microscope for the analysis of typically located PC soma cross-sectional area. We used thionin staining to quantify typically located PC soma cross-sectional area because calbindin labeling did not reliably fill the somata of typically located PCs, particularly in the portions of lobule VI adjacent to lobule IV/V. Typically located PC soma cross-sectional area measurements were performed in ImageJ. First, in ImageJ, the image scale was calibrated by measuring the length of the scale bar (known distance) to convert distance measured in pixels to microns. In total, we measured 24 PC somata per mouse. We selected six consistent locations within lobule VI to measure typically located PC soma cross-sectional area, selecting 4 PCs to measure from in each area to consider variability in soma size across the lobule. A PC's soma cross-sectional area was only quantified if the PC displayed a primary proximal dendrite in attempt to capture the maximal cross-sectional area of a PC. A PC's soma cross-sectional area was measured by drawing a polygon around the entire soma in ImageJ.

Measurements were performed by raters blinded to genotype. The 24 typically located PC somata cross-sectional area measurements were averaged within a mouse and resulted in one data point per mouse. Additionally, typically located PC somata cross-sectional areas were measured in ImageJ using confocal microscopy to verify that typically located PC somata cross-sectional areas measured via thionin were not significantly different from the same typically located PC somata cross-sectional areas measured via calbindin on the confocal.

Ectopic Purkinje Cell Cross-Sectional Soma Area

Using the same immuno-labeled sagittal sections from ectopic PC counts, three ectopic PCs in the granule layer of lobule VI per 16p11.2$^{dp/+}$ mouse were imaged at 40X with a zoom factor of 1.5 and resolution of 2048 x 2048 on a Leica SPE Confocal microscope. Thionin staining was not used to quantify ectopic PC soma cross-sectional area because ectopic PCs in the granule layer were indistinguishable from granule cells by thionin staining. Additionally, a confocal microscope was used to capture ectopic PCs instead of an epifluorescent microscope to reduce light scattering, which artificially inflates calbindin-labeled PC soma cross-sectional area. Ectopic PC soma cross-sectional area measurements were performed in ImageJ. First, the image scale was calibrated and the cross-sectional area of three ectopic PC somata previously identified in 16p11.2$^{dp/+}$ mice were measured by drawing a polygon around the entire soma in ImageJ. The three ectopic PC soma cross-sectional area measurements were averaged within a mouse and resulted in one data point per mouse.

Typically Located Purkinje Cell Density

The same immuno-labeled sagittal sections from ectopic PC counts were imaged at 4X to capture the entirety of lobule IV/V and lobule VI on an Olympus IX83 fluorescence microscope for the analysis of typically located PC density. Cell counts were performed in ImageJ by raters blinded to genotype. First, the image scale was calibrated. typically located PC counts were performed by adding a line in ImageJ across the PC monolayer, acquiring the distance of line in microns, and counting the number of PCs that spanned the line. This method was repeated in five consistent locations in lobule IV/V and lobule VI for each mouse section. In some sections, the PC somata were not reliably filled by calbindin in portions of lobule VI adjacent to lobule IV/V. To ensure accurate cell counts in these areas, we used thionin stained sections. The cell counts were divided by the length of the line

in microns to acquire a density measurement. Each of the five typically located PC density measurements were averaged to obtain one value per mouse.

Molecular Layer Interneuron Counts

One immuno-labeled sagittal section per mouse was imaged at 4X to capture the entirety of lobule IV/V and lobule VI in the cerebellar vermis on an Olympus IX83 fluorescence microscope for MLI cell counts. Cell counts were performed in ImageJ. First, in ImageJ, the image scale was calibrated and the DAPI and parvalbumin channel images (1 image and 1 section per mouse) were manually thresholded in ImageJ. The purpose of thresholding was to create a binary, such that DAPI+ and parvalbumin+ cells could be quantified in a semi-automated fashion. By assigning background fluorescence as a value of "0" and DAPI+ or parvalbumin+ cells assigned a fluorescence value of "1", ImageJ quantified pixels with a value of "1" as a DAPI+ or parvalbumin+ cell. After thresholding, polygons were drawn in the molecular layer using the DAPI channel and copied into the parvalbumin channel to maintain the same polygon size. The polygon area was then measured in mm^2 using Image J. The ImageJ "analyze particles" function was used to count both DAPI+ and parvalbumin+ cells within the polygon, using the following parameters: size: 2- infinity (pixel units) and circularity: 0.00-1.00. This created regions of interest for each cell, and cell counts were recorded for both channels. DAPI+ and parvalbumin+ cell counts were converted into units of cells per mm^2 using the following equation:

$$\frac{(DAPI + \ or\ Pvalb + \ cell\ counts\ )}{(Polygon\ area\ in\ mm^2)} = DAPI + \ or\ Pvalb + \ cells\ per\ mm^2$$

Thresholding and analysis were performed by raters blinded to genotype. MLI counts were performed for both lobule IV/V and VI. Five polygons per lobule in consistent locations across each mouse section were used to capture the entire molecular layer of each lobule. The cell counts for each polygon were then averaged and resulted in one value per mouse.

To determine if the distribution of DAPI+/parvalbumin+ MLIs was altered based on their locations within the molecular layer, we used a modified version of the procedure described above. Instead of drawing polygons spanning the entire width of the molecular layer, the thickness of the molecular layer of lobule VI was measured in ImageJ and the length divided to create inner 1/3 and outer 2/3 polygons (ten polygons in total per mouse). We used

this anatomical distinction to operationally define and distinguish between early and late-born MLIs. Early born MLIs reside largely in the inner 1/3 of the molecular layer (defined as basal 1/3 MLIs), while later-born MLIs reside mostly in the outer 2/3 of the molecular layer (defined as apical 2/3 MLIs)[1,2].

Behavioral procedures

Erasmus Ladder

The Erasmus Ladder task (Noldus, Wageningen, The Netherlands) is a test of motor function and motor associative learning that relies on the cerebellum[3]. It is made up of two enclosed boxes separated by a ladder, with a row of high rungs for the animals to efficiently cross the ladder, and a second row of lower rungs that record missteps in between the high rungs. In the first four days of training, animals are tested for abnormalities in gait adaptation, motor coordination, and associative learning. In the last four days of training, mice are tested for abnormalities in tone-cued cerebellar-dependent associative motor learning. Prior to Erasmus Ladder, all mice used in the behavior cohorts were weighed.

Mice were trained to traverse the Erasmus Ladder for 42 trials per day for four consecutive days. Trials consisted of one run across the ladder into the goal box and were separated by a random inter-trial interval ranging from 11-20 seconds. In the first four days of training, a bright light cue indicated the start of a trial. Animals that did not leave the starting box following 3 seconds of light cue stimulus experienced a tailwind of pressurized air to motivate them to cross the ladder. The apparatus measured the animal's steps across a discontinuous ladder and detected errors for each trial. Data were analyzed for the following: number of trials the animal left on light cue, number of trials the animal left on air cue, number of trials the animal went onto the ladder before a cue was given, frequency of the animal to return to the goal box, number of trials the animals refused to leave goalbox, percentage of missteps (lower rung steps/steps on ladder), percentage of correct long steps (skipping at least one high rung between steps), percentage of correct short steps (using consecutive high rungs). Post-perturbation step time was used to assess the baseline step time of an animal.

On the last four days of training, mice are tested for abnormalities in tone-cued cerebellar dependent associative motor learning. Mice were conditioned using a 75 decibel (dB) tone (CS) to anticipate the elevation of

a ladder rung (US), also called the perturbation, in which the mice had to learn to time their jump in accordance with the auditory cue. Data were analyzed for time the animal spent between steps immediately preceding the perturbation (pre-perturbation) and steps immediately following the perturbation (post-perturbation) for the last 5-8 days of the task. Learning is shown when animals spend less time on the pre and post perturbation step following consecutive days of training.

Given that 16p11.2$^{dp/+}$ mice display a startle response to auditory stimulus in PPI, this would suggest they have unimpaired hearing, and this permits the assessment of a variety of behaviors while mice learned to cross the Erasmus Ladder, including gait adaptation (learning to use longer steps to cross the ladder), motor coordination (missteps), cerebellar associative learning (learning to time a jump for an auditory cue) and associative learning (ability to learn visual and sensory start cues).

Eyeblink Conditioning

Delay eyeblink conditioning is a classical conditioning paradigm where eyelid closure, as an unconditioned response, initially occurs after the onset of the unconditioned stimulus (an air puff to the eye). Through repeated exposures to a conditioned stimulus (a light) and the air puff, adaptively timed eyelid closure begins to precede the air puff, resulting in a conditioned eyeblink response.

Prior to eyeblink conditioning, adult mice were anesthetized with isoflurane and placed into a stereotaxic frame. A heating pad was used to maintain body temperature during surgery. After the onset of anesthesia, 1 mg/mL of meloxicam per 20g of the animal's weight was administered for post-operative analgesia. The head was leveled, and a midline incision was made to expose the skull. The skin and underlying fascia was removed with tweezers and a cotton tipped applicator soaked in 70% ethanol. Once the skin was removed and the skull was dry, two small holes were drilled ~2 mm caudal and lateral to bregma. Self-tapping machine screws were secured to the skull before centering a stainless steel headplate between the screws. The screws and headplate were fixed to the skull with Metabond cement (Parkell, Inc). After the cement dried, mice were returned to their home cage to recover under a heat lamp.

Before the start of an eyeblink conditioning session, each mouse was lightly anesthetized with isoflurane in an induction chamber. All eyeblink sessions were conducted inside an operant chamber with white noise (65 dB) generated through a speaker. After the onset of anesthesia, the headplate was fastened to a pair of machined rods with 2-56 machine screws. Each rod was attached to posts that surrounded a low friction freely moving Styrofoam cylinder. The height of the machined rods was adjusted so that each mouse could walk freely on the cylinder after the headplate was secured. A high-speed (200 frames/s) monochrome camera (Allied Vision or Basler) and infrared illumination were attached to a different post and were directed toward the right eye. For the air puff unconditioned stimulus (US) (20-30 PSI, 20-30 ms), plastic tubing connected to an air compressor/regulator was attached to a 23-gauge needle housed in an adjustable case. The 23-gauge needle was adjusted so the final position was 3 mm from the right eye and directed at the middle of the cornea. The intensity of the air puff US was 6-8 PSI measured at the end of the needle. Time of actual puff delivery was corrected to account for the delays introduced by the solenoid and tubing, as measured at the end of the needle by a silicon pressure sensor (Honeywell HSCSANN 150PG2A3, DigiKey). The conditioned stimulus (CS) was a blue light LED mounted on a post in front of the mouse and the timing of the CS and US was controlled by a custom Arduino-based device[4,5].

Mice received two habituation sessions prior to eyeblink conditioning. Before the habituation (and eyeblink conditioning) sessions, mice were initially lightly anesthetized with isoflurane in an induction chamber. After breathing slowed, they were transferred to the operant chamber and secured over the cylinder. The habituation sessions allowed mice to move freely on the cylinder while head-fixed for one hour without presentation of the CS or US. Following habituation, mice were given ten daily eyeblink conditioning sessions (up to 100 trials per day) consisting of 10 trial blocks (9 CS/US paired, 1 CS alone trial) with an interstimulus interval of 400 ms. After mice were secured over the cylinder and had recovered from anesthesia, they received presentations of the air puff US to calibrate the reflexive eyelid closure. Eyelid movements were measured frame-by-frame by calculating the area of the eye visible within a custom fit elliptical region of interest. Raw pixel counts were normalized into units of fraction of eyelid closure (FEC) ranging from fully open (0) to fully closed (1). Once full eyelid closure was calibrated the eyeblink conditioning session was started.

Eyelid traces for each trial were extracted from the high-speed video files using the “pixel area” (FEC) algorithm. For paired trials, a conditioned response (CR) was operationally defined as an increase in eyelid amplitude during the final 300 ms of the CS that was greater than 10% of the unconditioned response amplitude to the air puff (full eyelid closure) for each trial. For CS alone trials, a CR was operationally defined as an increase in eyelid amplitude 100 ms after CS onset that was greater than 10% of the session average unconditioned response amplitude. CR onset latency was defined as the first time point when eyelid amplitude reached 10% of the unconditioned response amplitude for a given trial (paired trials) or 10% of the session average unconditioned response (CS alone trials). CR maximum amplitude was defined as the largest eyelid amplitude value during the interstimulus interval. For paired trials, data from the final 300 ms of the CS was used for CR onset latency. For CS alone trials, all position data collected after CS onset were used for CR percentage, amplitude, and latency analysis.

Activity Monitoring

Adult mice approximately 3 months of age were placed into specially designed home cages (Columbus Instruments) with infrared photobeams to record locomotor activity. The configuration of the beams track activity to generate a record of movement across the XY (horizontal) and Z (vertical) axes, quantified as beam breaks. Mice lived in the modified home cages for two weeks (the first week was used as habituation and was not analyzed). The second week of data was analyzed for activity. Mice were provided food and water ad libitum. Activity monitoring was performed under a regular light cycle with lights on/off at 0900/2100 DST (0800/2000 non-DST). Two home cages did not record Z activity, resulting in loss of Z activity data from one WT and one $16p11.2^{dp/+}$ mouse.

Prepulse Inhibition

Mice were placed in an isolation cabinet and restrained in a clear acrylic cylinder. The tremble response of the animal was measured via an accelerometer underneath the chamber and the testing apparatus consisted of a startle response box (SR-LAB, San Diego Instruments). Mice were habituated to the testing chamber for 10 minutes with a consistent background white noise level of 65 dB, which continued for the duration of the experiment. Each mouse underwent 64 trials. The first and last 6 trials (Block I and Block III) consisted of solely pulse alone trials to

verify that mice were not acclimating to the startle pulse over the experiment. All trials were presented with a randomly spaced inter-trial interval ranging from 7 to 15 seconds. The startle pulse was set to 120 dB and the prepulse intensities were set to 5, 10, and 15 dB above the background noise in the testing room. The startle response was recorded in millivolts in SR-LAB software. Percent PPI was calculated by normalizing the startle response of the pulse alone trials from block II using the following equation:

$$PPI\,\% = \frac{(startle\ response\ for\ pulse\ alone - startle\ response\ for\ pulse\ with\ prepulse)}{startle\ response\ for\ pulse\ alone} \cdot 100$$

Statistics and Supplemental Results

General

All behavioral data except delay eyeblink conditioning were analyzed to assess for sex as a biological variable. Data were graphed and analyzed using GraphPad Prism 10.6 (GraphPad Software, San Diego, CA) and R (R 4.1.1, emmeans 1.7.0, lme4 1.1.27.1, lmerTest 3.1-3, effectsize 0.5) and are graphically represented as mean ± standard error of the mean (SEM) for each group. Normality tests were run to determine the appropriate statistical method for analysis of each experiment. Outlier tests were run using ROUT with a Q = 0.2% (this resulted in one WT and one 16p11.2$^{dp/+}$ mouse being excluded from activity monitoring analysis). Data were analyzed using the statistical test noted in results and figure legends. Results were considered significant when $p < 0.05$ (denoted in all graphs as follows: *$p < 0.05$; **$p < 0.01$).

Histological Data

A nested t test was used to detect group differences for ectopic PC counts. A nonparametric, unpaired t test (Mann-Whitney test) was used to detect group differences for typically-located PC soma cross-sectional area. An unpaired t test was used to detect group differences for ectopic PC soma cross-sectional area. A two-way ANOVA was used to detect group differences for typically-located PC density. Multiple unpaired t tests using the Holm-Sidak method were used to compare DAPI+ and parvalbumin+ MLI cell counts. In lobule VI, multiple unpaired t tests using the Holm-Sidak method were used to compare DAPI+ basal 1/3, DAPI+ apical 1/3, parvalbumin+ basal 1/3, and parvalbumin+ apical 2/3 MLI cell counts in WT and 16p11.2$^{dp/+}$ mice.

Erasmus Ladder

Erasmus Ladder data were analyzed via linear mixed-effects modeling in RStudio with the ImerTest and emmeans packages and graphed in GraphPad Prism 10.6.

Eyeblink Conditioning

Data were organized and analyzed in GraphPad Prism 10.6 using Linear Mixed Effects Model (Type III). Group data was graphed in Prism and edited using Adobe Illustrator. Tukey's (LSD) was used for post-hoc tests.

CR Amplitude

CR amplitude during CS+US combined trials was analyzed using a mixed effects model (REML) with Geisser-Greenhouse correction (ε = 0.3249). There was a significant main effect of session ($F(2.924, 38.02) = 5.59$, $p = 0.003$), indicating that CR amplitude changed across sessions. However, there was no significant main effect of genotype ($F(1, 13) = 0.76$, $p = 0.399$) and no significant genotype × session interaction ($F(2.924, 38.02) = 0.77$, $p = 0.516$). These results indicate no difference in CR amplitude during combined trials between WT and $16p11.2^{dp/+}$ mice.

CS alone trial analyses

CR percentage during CS alone trials was analyzed using a mixed effects model (REML) with Geisser-Greenhouse correction (ε = 0.3715). There was a significant main effect of session ($F(3.343, 43.46) = 18.59$, $p < 0.0001$), indicating that CR percentage changed across sessions in both groups. However, there was no significant main effect of genotype ($F(1, 13) = 0.32$, $p = 0.582$) and no significant genotype × session interaction ($F(3.343, 43.46) = 0.89$, $p = 0.461$). These results indicate no difference in CR percentage during CS alone trials between WT and $16p11.2^{dp/+}$ mice.

CR onset latency during CS alone trials was analyzed using a mixed effects model (REML) with Geisser-Greenhouse correction (ε = 0.4188). There was a significant main effect of session ($F(3.769, 42.30) = 3.67$, $p = 0.013$), but no significant main effect of genotype ($F(1, 13) = 1.15$, $p = 0.302$) and no significant genotype × session interaction ($F(3.769, 42.30) = 2.33$, $p = 0.075$). These results indicate no difference in CR onset latency during CS alone trials between WT and $16p11.2^{dp/+}$ mice.

CR amplitude during CS alone trials was analyzed using a mixed effects model (REML) with Geisser-Greenhouse correction ($\varepsilon = 0.3528$). There was no significant main effect of session ($F(3.175, 35.63) = 1.80$, $p = 0.162$), no significant main effect of genotype ($F(1, 13) = 0.26$, $p = 0.618$), and no significant genotype × session interaction ($F(3.175, 35.63) = 0.21$, $p = 0.899$). These results indicate no difference in CR amplitude during CS alone trials between WT and $16p11.2^{dp/+}$ mice.

Activity Monitoring

Data were analyzed for activity in 1h bins (hour-by-hour analysis) using a mixed design ANOVA with sex and genotype as the between-subjects variables and time as the within-subject variable in SPSS 29.0.1.0. When sphericity was violated, Greenhouse-Geisser corrected F-values were used. A multivariate analysis of variance (MANOVA) was used to analyze activity during the light cycle and the dark cycle. Sidak adjusted posthoc pairwise comparisons were used in all cases.

Prepulse Inhibition

All data from PPI were analyzed to assess for sex, genotype, decibel, and block differences.

References

1. Brown AM, Arancillo M, Lin T, et al. Molecular layer interneurons shape the spike activity of cerebellar Purkinje cells. *Sci Rep*. 2019;9(1):1742. doi:10.1038/s41598-018-38264-1

2. Wang WX, Lefebvre JL. Morphological pseudotime ordering and fate mapping reveal diversification of cerebellar inhibitory interneurons. *Nat Commun*. 2022;13:3433.

3. Giessen RSVD, Koekkoek SK, Dorp S van, et al. Role of Olivary Electrical Coupling in Cerebellar Motor Learning. *Neuron*. 2008;58(4):599-612. doi:10.1016/j.neuron.2008.03.016

4. Heiney SA, Kim J, Augustine GJ, Medina JF. Precise Control of Movement Kinematics by Optogenetic Inhibition of Purkinje Cell Activity. *The Journal of Neuroscience*. 2014;34(6):2321. doi:10.1523/JNEUROSCI.4547-13.2014

5. Heiney SA, Ohmae S, Kim OA, Medina JF. Single-Unit Extracellular Recording from the Cerebellum During Eyeblink Conditioning in Head-Fixed Mice. *Neuromethods*. 2018;134:39-71. doi:10.1007/978-1-4939-7549-5_3

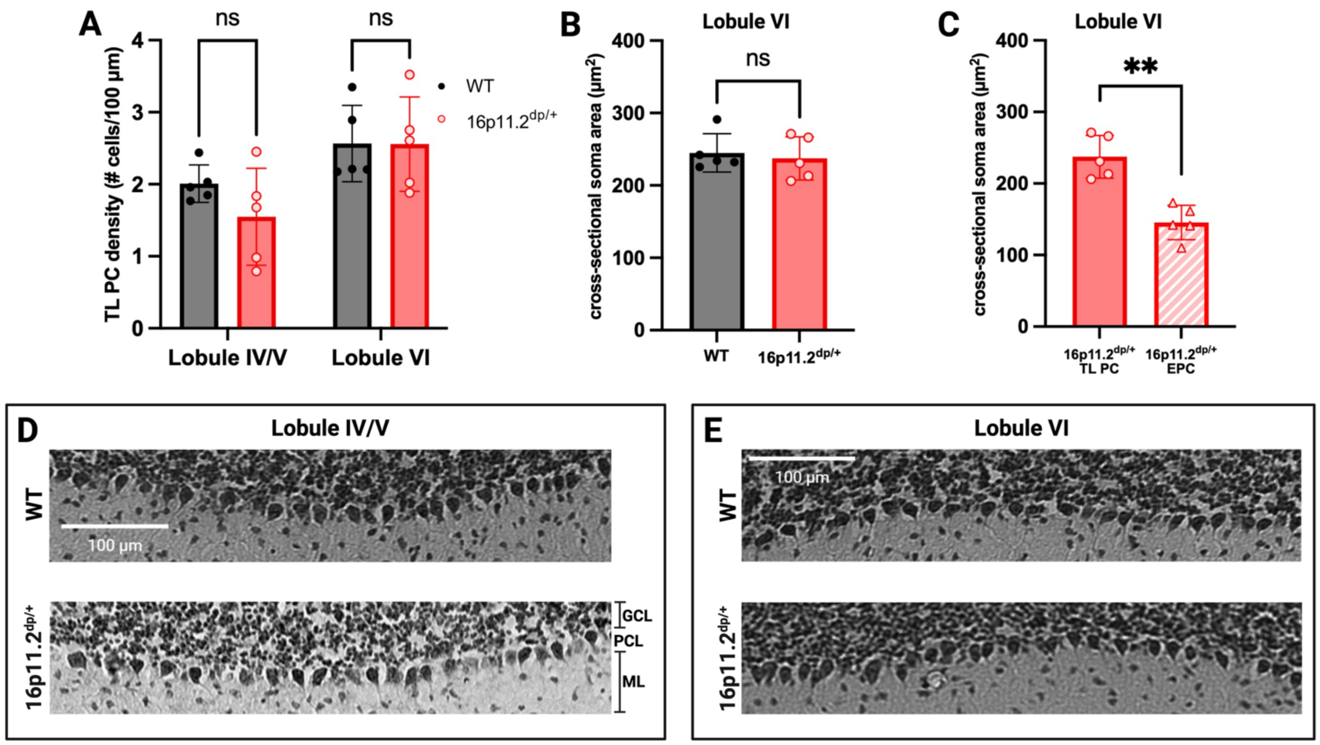

Supplementary Figure 1. Quantification of density and size of Purkinje cells. A. There were no differences in typically located Purkinje cell (TL PC) density in lobule IV/V or lobule VI (two-way ANOVA). **B.** No differences were found in the cross-sectional soma area of typically located Purkinje cells in lobule VI (Mann-Whitney test). **A-B:** Mean ± SD, n = 10 (WTs (black): n = 3 females, n = 2 males; 16p11.2$^{dp/+}$(red): n = 2 females, n = 3 males), age = 3.3-3.5 months old. Each dot represents the mean value from each mouse. **C.** Typically located Purkinje cells (TL PC) were significantly larger than ectopic Purkinje cells (EPC) (unpaired t test, mean ± SD, n = 5 (16p11.2$^{dp/+}$: n = 2 females, n = 3 males), age = 3.3-3.5 months old). Each dot represents the mean value from each mouse. **D.** Representative image of thionin-stained section of cerebellar cortex from lobule IV/V in WT and 16p11.2$^{dp/+}$ mice. GCL = granule cell layer, PCL = Purkinje cell layer, ML = molecular layer. Scalebar = 100µm. **E.** Representative image of thionin-stained section of cerebellar cortex from lobule VI in WT and 16p11.2$^{dp/+}$ mice. scalebar = 100µm. Created in BioRender. Williams lab, A. (2026) https://BioRender.com/xr3fz4i.

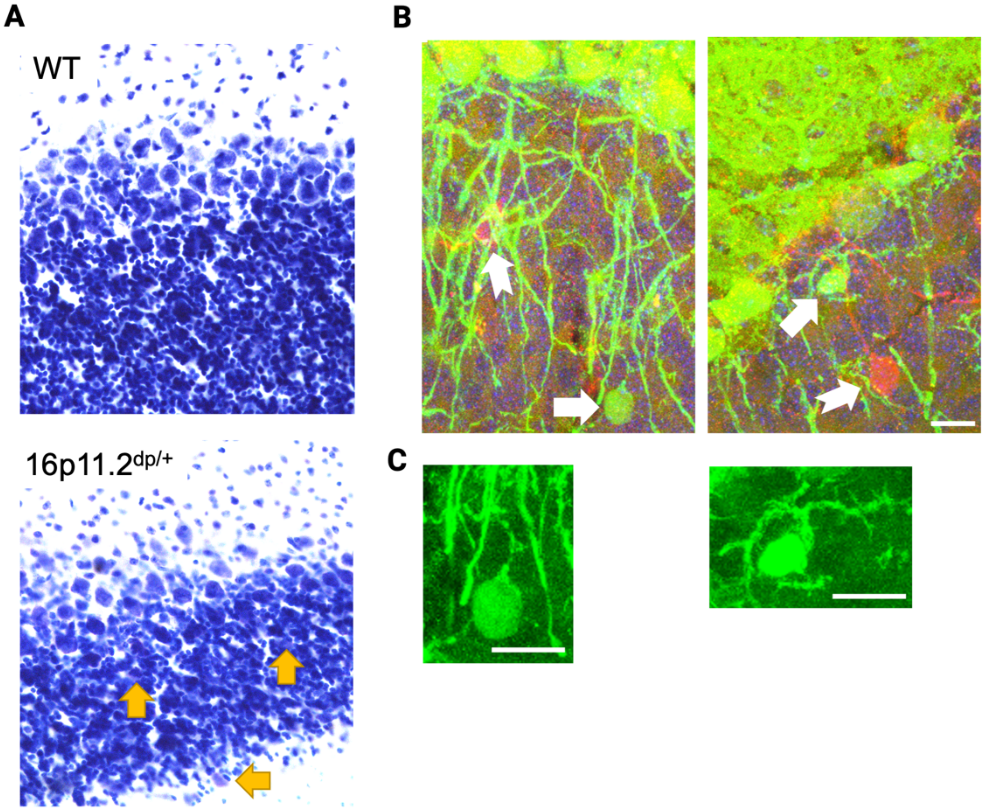

Supplementary Figure 2. Ectopic Purkinje cells are distinct from Golgi cells. A. Thionin-stained coronal sections of 13-month-old WT (top) and 16p11.2$^{dp/+}$ (bottom) cerebellum. Orange arrows indicate ectopic Purkinje cells in the granule layer. **B.** Sagittal sections of 16p11.2$^{dp/+}$ cerebellar vermis at 5.1 months of age. Solid arrows indicate ectopic Purkinje cells labeled with calbindin (green) and notched arrows show Golgi cells labeled with neurogranin (red). Scalebar = 15µm. **C.** Ectopic Purkinje cells labeled with calbindin from panel B are shown in greater detail. Scalebar = 15µm.

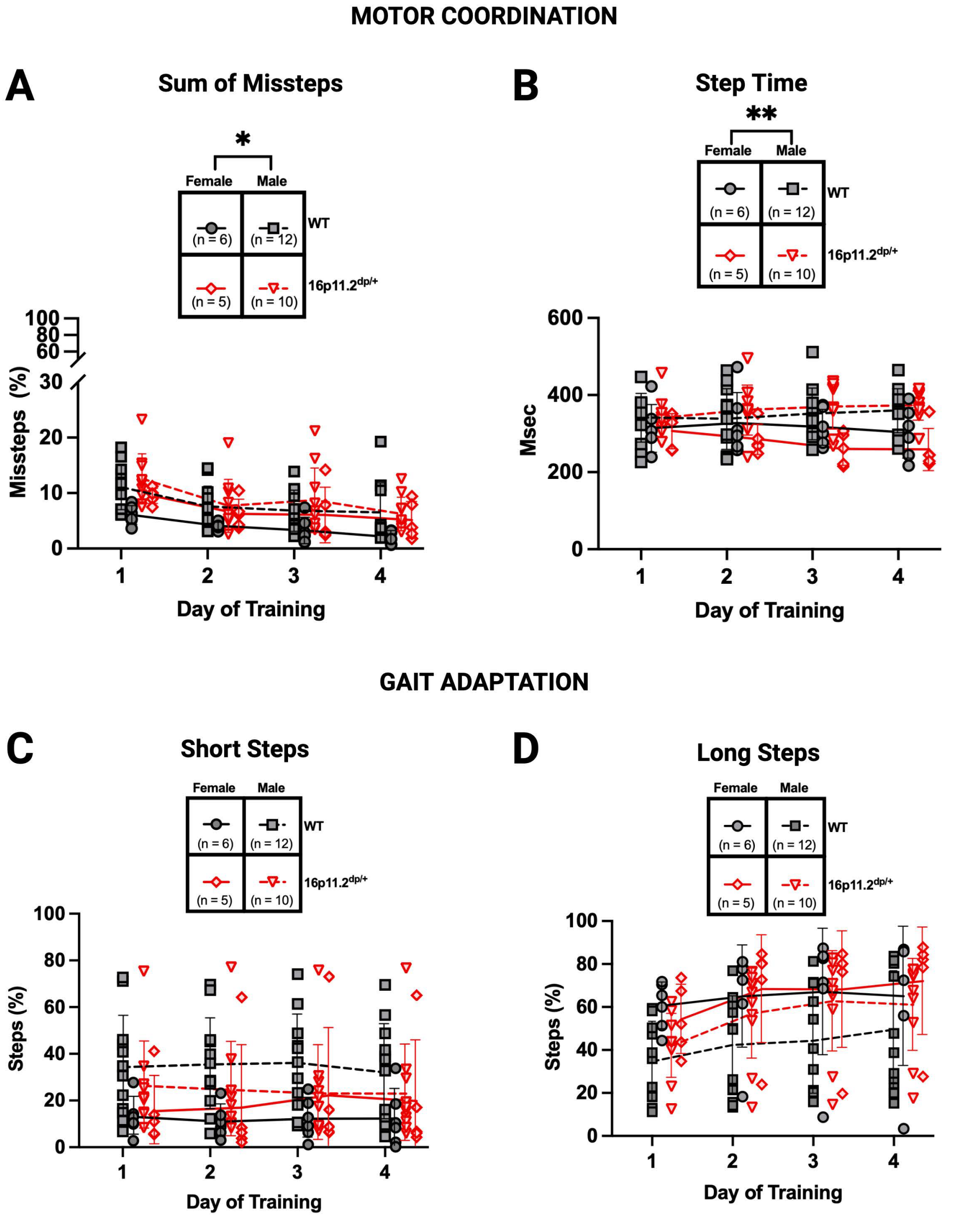

Supplementary Figure 3. Typical motor coordination and gait adaptation in 16p11.2$^{dp/+}$ mice. A. No differences were found in percentage of missteps used between WT and 16p11.2$^{dp/+}$ littermates. However, female mice displayed fewer missteps than male mice (F (1,29) = 7.10, p = 0.013). **B.** No differences were found in step time between WT and 16p11.2$^{dp/+}$ littermates. However, female mice were faster than male mice (F (1,29) = 9.51, p = 0.0045), and their step time increased more than male mice on days 3 (t ratio (52.8) = -3.31, p = 0.0017) and 4 (t ratio (52.3) = -3.86, p = 0.0003) (indicated on graph with asterisk, asterisk indicates significant difference). **C.** No differences were found in the percentage of short steps used. **D.** All mice increased their percentage of long steps used as days of training progressed (main effect of day not indicated on graph) (F (3,86) = 17.89, p < 0.0001). However, there were no sex or genotype

differences. **A-D**: Linear mixed effects modeling, Type III ANOVA Satterthwaite's method. Mean ± SD, n = 33 (WTs: n = 6 females, n = 12 males; 16p11.2$^{dp/+}$: n = 5 females, n = 10 males), mice were between 3.3-3.5 months old at the time of the experiment. Created in BioRender. Williams lab, A. (2026) https://BioRender.com/75xjseb.

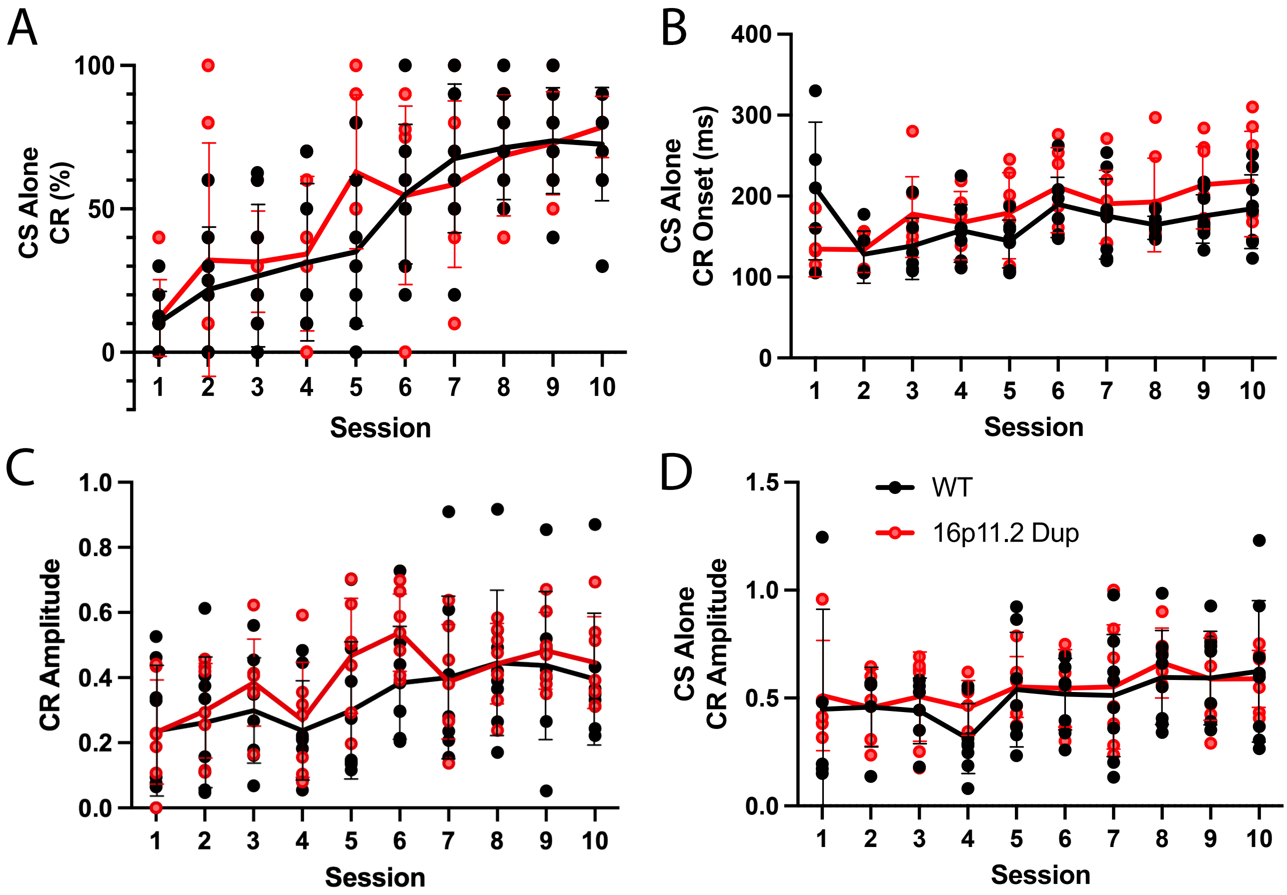

Supplementary Figure 4. CS alone trial analyses and CR amplitude in WT and 16p11.2$^{dp/+}$ mice during EBC training. No significant genotype differences were observed between 16p11.2$^{dp/+}$ mice and WT littermates for CS alone CR% (A), CS alone CR onset latency (B), CR amplitude during CS+US combined trials (C), or CS alone CR amplitude (D). Mixed effects model (REML) with Geisser-Greenhouse correction, Type III fixed effects. Mean ± SD, *n* = 15 (WT: *n* = 3 females, *n* = 8 males; 16p11.2$^{dp/+}$ mice: *n* = 5 females, *n* = 2 males), age = 4.8–6.8 months.

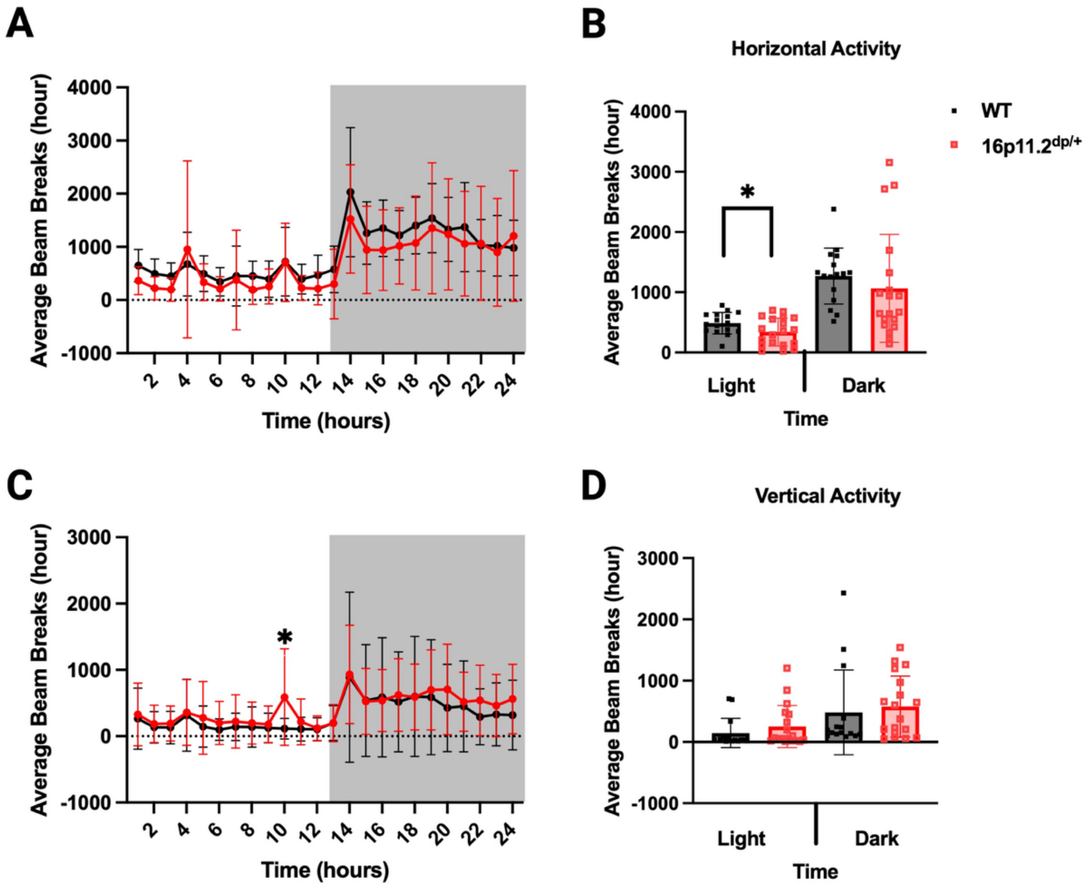

Supplementary Figure 5. Activity monitoring in 16p11.2$^{dp/+}$ mice. A. No differences were found in average horizontal (XY) activity hour-by-hour between WT and 16p11.2$^{dp/+}$ littermates. Gray box indicates the dark phase of the day (lights off). Time shown in zeitgeber hours where lights on is 0-13 hours and lights off is 13-24 hours. **B.** 16p11.2$^{dp/+}$ mice showed less spontaneous horizontal activity overall during the light phase than WT mice ($p = 0.043$), although no differences were noted during the dark phase. **C.** Average vertical (Z) activity differed only at hour 10 between WT and 16p11.2$^{dp/+}$ littermates ($F(1,29) = 5.45$, $p = 0.026$). **D.** No differences were observed in average vertical (Z) activity overall during the light or the dark phase. **A-B**: General linear model for hour-by-hour; MANOVA for light vs. dark. Mean ± SD, $n = 35$ (WTs: $n = 8$ females, $n = 8$ males; 16p11.2$^{dp/+}$: $n = 11$ females, $n = 8$ males). **C-D**: General linear model for hour-by-hour; MANOVA for light vs. dark. Mean ± SD, $n = 33$ (WTs: $n = 8$ females, $n = 7$ males; 16p11.2$^{dp/+}$: $n = 11$ females, $n = 7$ males). Created in BioRender. Williams lab, A. (2026) https://BioRender.com/2qpd065.

HISTOLOGY

Cell Type	Assay	Measurement	Stain method	Cell type ID	n	Age	Statistics
PCs	Granule layer EPC counts	Manual cell counts and averaged	IHC	calb+/pvalb+ and in GL	WTs: n = 5 (3F, 2M)<br>dp/+: n = 5 (2F, 3M)	3.1-3.5 mo	Nested t test
PCs	TL PC soma CSA	Average soma CSA – measured 4 TL PCs in 6 consistent locations/lobule	Thionin staining	Size and in PC monolayer	WTs: n = 5 (3F, 2M)<br>dp/+: n = 5 (2F, 3M)	3.1-3.5 mo	Mann-Whitney test
PCs	EPC soma CSA vs. TL PC in 16p11.2dp/+	Average soma CSA	Ectopic PCs: IHC (confocal)<br>TL PCs: Thionin staining	Ectopic PCs: calb+/pvalb+ and in GL<br>TL PCs: Size and in PC monolayer	dp/+: n = 5 (2F, 3M)	3.1-3.5 mo	Unpaired t test
PCs	TL PC density	Average number of PCs/distance (density) – measured in 5 consistent locations/lobule	IHC	calb+/pvalb+ and in PC monolayer	WTs: n = 5 (3F, 2M)<br>dp/+: n = 5 (2F, 3M)	3.1-3.5 mo	2-way ANOVA
MLIs	MLI counts	Average number of DAPI+ cells / 0.1mm^2– measured in 5 consistent locations/lobule<br>Average number of pvalb+ cells/0.1mm^2 – measured in 5 consistent locations/lobule	IHC	DAPI+ and in ML<br>DAPI+/pvalb+ and in ML	WTs: n = 5 (3F, 2M)<br>dp/+: n = 5 (2F, 3M)	3.1-3.5 mo	Multiple unpaired t tests (Holm-Sidak method)
MLIs	Basal 1/3 MLI counts	Average number of DAPI+ cells / 0.1mm^2– measured in 5 consistent locations/lobule<br>Average number of pvalb+ cells/0.1mm^2 – measured in 5 consistent locations/lobule	IHC	DAPI+ and in basal 1/3 of ML<br>DAPI+/pvalb+ and in basal 1/3 of ML	WTs: n = 5 (3F, 2M)<br>1dp/+: n = 5 (2F, 3M)	3.1-3.5 mo	Multiple unpaired t tests (Holm-Sidak method)
MLIs	Apical 2/3 MLI counts	Average number of DAPI+ cells/0.1mm^2– measured in 5 consistent locations/lobule<br>Average number of pvalb+ cells/0.1mm^2 – measured in 5 consistent locations/lobule	IHC	DAPI+ and in apical 2/3 of ML<br>DAPI+/pvalb+ and in apical 2/3 of ML	WTs: n = 5 (3F, 2M)<br>dp/+: n = 5 (2F, 3M)	3.1-3.5 mo	Multiple unpaired t tests (Holm-Sidak method)

ANOVA table

Assay	Factors	SS	DF	MS	F(DFn, DFd)	Dif. Between Means	SE of Difference	P-value	Figure
Granule layer EPC counts	N/A	N/A	N/A	N/A	N/A	N/A	N/A	N/A	N/A
TL PC soma CSA	N/A	N/A	N/A	N/A	N/A	N/A	N/A	N/A	N/A
EPC soma CSA vs. TL PC in 16p11.2dp/+	N/A	N/A	N/A	N/A	N/A	N/A	N/A	N/A	N/A
TL PC density	Lobule x Genotype	0.2547	1	0.2547	F(1,8) = 0.5705	N/A	N/A	0.4717	S1A
	Lobule	**3.068**	**1**	**3.068**	**F(1,8) = 6.8730**	**-0.7834**	**0.2988**	**0.0306**	
	Genotype	0.2748	1	0.2748	F(1,8) = 1.631	0.2344	0.1836	0.2374	
	Subject	1.348	8	0.1685	F(8,8) = 0.3775	N/A	N/A	0.9051	
	Residual	3.571	8	0.4464	N/A	N/A	N/A	N/A	
MLI counts	N/A	N/A	N/A	N/A	N/A	N/A	N/A	N/A	N/A
Basal 1/3 MLI counts	N/A	N/A	N/A	N/A	N/A	N/A	N/A	N/A	N/A
Apical 2/3 MLI counts	N/A	N/A	N/A	N/A	N/A	N/A	N/A	N/A	N/A

Lobule IV/V

Assay	Dif. Between Means	SE of difference	Std Error	t ratio	t-stat	DF	p-value	Figure
Granule layer EPC counts	1.95	N/A	± 2.181	N/A	0.8941	8	0.3974	1A
TL PC soma CSA	N/A	N/A	N/A	N/A	N/A	N/A	N/A	N/A
EPC soma CSA vs. TL PC in 16p11.2dp/+	N/A	N/A	N/A	N/A	N/A	N/A	N/A	N/A
TL PC density	N/A	N/A	N/A	N/A	N/A	N/A	N/A	N/A
MLI counts	DAPI+ = -99.65<br>pvalb+ = 186.00	DAPI+ = 150.90<br>pvalb+ = 96.33	N/A	DAPI+ = 0.6605<br>pvalb+ = 1.931	N/A	8<br>8	DAPI+ = 0.5275<br>pvalb+ = 0.1711	2A
Basal 1/3 MLI counts	N/A	N/A	N/A	N/A	N/A	N/A	N/A	N/A
Apical 2/3 MLI counts	N/A	N/A	N/A	N/A	N/A	N/A	N/A	N/A

Lobule VI

Assay	Dif. Between Means	SE of difference	Std Error	t ratio	t-stat	DF	Actual Difference	U-value	p-value	Figure
Granule layer EPC counts	**6.250**	N/A	**± 1.451**	N/A	**4.308**	**8**	N/A	N/A	**0.0026**	1B
TL PC soma CSA	N/A	N/A	N/A	N/A	N/A	N/A	-3.304	9	0.5476	S1B
EPC soma CSA vs. TL PC in 16p11.2dp/+	**-91.85**	N/A	**± 17.13**	N/A	**5.363**	**8**	N/A	N/A	**0.0007**	S1C
TL PC density	N/A	N/A	N/A	N/A	N/A	N/A	N/A	N/A	N/A	N/A
MLI counts	DAPI+ = -107.10<br>**pvalb+ = 462.60**	DAPI+ = 138.40<br>**pvalb+ = 77.23**	N/A	DAPI+ = 0.7737<br>**pvalb+ = 5.9910**	N/A	8<br>**8**	N/A	N/A	DAPI+ = 0.4614<br>**pvalb+ = 0.0007**	2B
Basal 1/3 MLI counts	DAPI+ basal 1/3 = -62.95<br>pvalb+ basal 1/3 = 10.85	DAPI+ basal 1/3 = 173.70<br>pvalb+ basal 1/3 = 119.80	N/A	DAPI+ basal 1/3 = 0.3624<br>pvalb+ basal 1/3 = 0.0906	N/A	8<br>8	N/A	N/A	DAPI+ basal 1/3 = 0.9795<br>pvalb+ basal 1/3 = 0.9951	2D
Apical 2/3 MLI counts	DAPI+ apical 2/3 = -15.36<br>**pvalb+ apical 2/3 = 413.60**	DAPI+ apical 2/3 = 213.20<br>**pvalb+ apical 2/3 = 86.25**	N/A	DAPI+ apical 2/3 = 0.0721<br>**pvalb+ apical 2/3 = 4.795**	N/A	8<br>**8**	N/A	N/A	DAPI+ apical 2/3 = 0.9951<br>**pvalb+ apical 2/3 = 0.0054**	2D

Supplementary Table 1. Statistical results for histology experiments. Significant differences are noted in light orange boxes with bold text.

BEHAVIOR											
Task	Assay	Measurement	n	Age	Statistics	Genotype	Sex	Day/Session/Block	Decibel	Interaction	Figure
Erasmus Ladder	Motor Coordination	% Missteps (% L-L steps)	WT: n = 18 (6F, 12M);<br>dp/+: n = 15 (5F, 10M)	3.3-3.5 mo	Type III ANOVA Satterwaite's Method	F(1,29) = 2.5194<br>SS = 19.810<br>MS = 19.812<br>p = 0.1233	F(1,29) = 7.1035<br>SS = 55.860<br>MS = 55.860<br>estimate = -3.1<br>SE = 1.16<br>df = 28.9<br>t ratio = -2.665<br>p = 0.0125<br>F < M	F(3,86) = 15.7412<br>SS = 371.350<br>MS = 123.785<br>p = 0.0001<br>Reduced by day<br>Day 1 vs. 2:<br>estimate = 3.385<br>SE = 0.735<br>df = 86<br>t ratio = 4.604<br>p = 0.0001<br>Day 1 vs. 3:<br>estimate = 3.600<br>SE = 0.738<br>df = 86.1<br>t ratio = 4.875<br>p < 0.0001<br>Day 1 vs. 4:<br>estimate = 4.809<br>SE = 0.735<br>df = 86<br>t ratio = 6.541<br>p < 0.0001<br>Day 2 vs. 4:<br>estimate = 1.424<br>SE = 0.735<br>df = 86<br>t ratio = 1.937<br>p = 0.2205	N/A	No	S3A
		Step Time (Cross time)	WT: n = 18 (6F, 12M);<br>dp/+: n = 15 (5F, 10M)	3.3-3.5 mo	Type III ANOVA Satterwaite's Method	F(1,29) = 0.4170<br>SS = 549.0<br>MS = 549.0<br>p = 0.5235	F(1,29) = 9.5058<br>SS = 12513.2<br>MS = 12513.2<br>estimate = -58.6<br>SE = 19<br>df = 28.9<br>t ratio = -3.083<br>p = 0.0045<br>F > M	F(3,86) = 0.0752<br>SS = 297.1<br>MS = 99<br>p = 0.9732	N/A	Sex x Day<br>F(3,86) =3.6530<br>SS = 14426.2<br>MS = 4808.7<br>p = 0.0156<br>F faster by day<br>Day 1 - Female vs. Male:<br>estimate = -30.9<br>SE = 22.3<br>df = 52.3<br>t ratio = -1.385<br>p = 0.1718<br>Day 2 - Female vs. Male:<br>estimate = -43.5<br>SE = 22.3<br>df = 52.3<br>t ratio = -1.950<br>p = 0.0565<br>Day 3 - Female vs. Male:<br>estimate = -74.1<br>SE = 22.4<br>df = 52.8<br>t ratio = -3.312<br>p = 0.0017<br>Day 4 - Female vs. Male:<br>estimate = -86<br>SE = 22.3<br>df = 52.3<br>t ratio = -3.859<br>p = 0.0003	S3B
	Gait Adaptation	% Short steps (step on all rungs)	WT: n = 18 (6F, 12M);<br>dp/+: n = 15 (5F, 10M)	3.3-3.5 mo	Type III ANOVA Satterwaite's Method	F(1,29) = 0.0541<br>SS = 1.347<br>MS = 1.347<br>p = 0.8177	F(1,29) = 3.7623<br>SS = 93.730<br>MS = 93.730<br>p = 0.0622	F(3,86) = 0.2906<br>SS = 21.715<br>MS = 7.238<br>p = 0.8321	N/A	No	S3C
		% Long steps (skipping rungs)	WT: n = 18 (6F, 12M);<br>dp/+: n = 15 (5F, 10M)	3.3-3.5 mo	Type III ANOVA Satterwaite's Method	F(1,29) = 0.7058<br>SS = 48.10<br>MS = 48.13<br>p = 0.4077	F(1,29) = 3.6680<br>SS = 250.10<br>MS = 250.11<br>p = 0.0654	F(3,86) = 17.8853<br>SS = 3658.70<br>MS = 1219.57<br>p < 0.0001<br>Increased by day<br>Day 1 vs. 2:<br>estimate = -10.314<br>SE = 2.17<br>df = 86<br>t = -4.764<br>p < 0.0001<br>Day 1 vs. 3:<br>estimate = -13.300<br>SE = 2.17<br>df = 86<br>t ratio = -6.115<br>p < 0.0001<br>Day 1 vs. 4:<br>estimate = -14.052<br>SE = 2.17<br>df = 86<br>t ratio = -6.490<br>p < 0.0001<br>Day 2 vs. 4:<br>estimate = -3.737<br>SE = 2.17<br>df = 86<br>t ratio = -1.726<br>p = 0.3166	N/A	No	S3D
	Associative Learning	Leaving on light cue (#trials days 1-4)	WT: n = 18 (6F, 12M);<br>dp/+: n = 15 (5F, 10M)	3.3-3.5 mo	Type III ANOVA Satterwaite's Method	F(1,29) = 4.9755<br>SS = 89.880<br>MS = 89.884<br>estimate = 4.67<br>SE = 2.09<br>df = 28.9<br>t ratio = 2.231<br>p = 0.0336<br>dp/+ < WTs	F(1,29) = 0.9314<br>SS = 16.830<br>MS = 16.825<br>p = 0.3425	F(3,86) = 14.3452<br>SS = 777.450<br>MS = 259.150<br>p < 0.0001<br>Increased by day<br>Day 1 vs. 2:<br>estimate = -2.67<br>SE = 1.11<br>df = 86<br>t ratio = -2.397<br>p = 0.0854<br>Day 1 vs. 3:<br>estimate = -5.13<br>SE = 1.12<br>df = 86.1<br>t ratio = -4.582<br>p = 0.0001<br>Day 1 vs. 4:<br>estimate = -6.86<br>SE = 1.11<br>df = 86<br>t ratio = -6.154<br>p < 0.0001<br>Day 2 vs. 3:<br>estimate = -2.46<br>SE = 1.12<br>df = 86.1<br>t ratio = -2.196<br>p = 0.1325<br>Day 2 vs. 4:<br>estimate = -4.19<br>SE = 1.11<br>df = 86<br>t ratio = -3.757<br>p = 0.0017<br>Day 3 vs. 4:<br>estimate = -1.73<br>SE = 1.12<br>df = 86.1<br>t ratio = -1.545<br>p = 0.4157	N/A	Genotype x Day<br>F(3,86) = 3.7700<br>SS = 204.320<br>MS = 68.105<br>p = 0.0136<br>dp/+ less on days 3 and 4<br>Day 1 - WT vs. dp/+:<br>estimate = 0.942<br>SE = 2.50<br>df = 55.4<br>t ratio = 0.377<br>p = 0.7078<br>Day 2 - WT vs. dp/+:<br>estimate = 3.683<br>SE = 2.50<br>df = 55.4<br>t ratio 1.474<br>p = 0.1462<br>Day 3 - WT vs. dp/+:<br>estimate = 6.000<br>SE = 2.51<br>df = 56<br>t ratio = 2.393<br>p = 0.0201<br>Day 4 - WT vs. dp/+:<br>estimate = 8.058<br>SE = 2.50<br>df = 55.4<br>t ratio = 3.225<br>p = 0.0021	3A
		Leaving on Air Cue (#trials days 1-4)	WT: n = 18 (6F, 12M);<br>dp/+: n = 15 (5F, 10M)	3.3-3.5 mo	Type III ANOVA Satterwaite's Method	F(1,29) = 4.6359<br>SS = 136.770<br>MS = 136.774<br>estimate = -5.27<br>SE = 2.45<br>df = 28.9<br>t ratio = -2.153<br>p = 0.0398<br>dp/+ > WTs	F(1,29) = 1.6332<br>SS = 48.190<br>MS = 48.186<br>p = 0.2114	F(3,86) = 5.2782<br>SS = 467.170<br>MS = 155.723<br>p = 0.0022<br>Decreased by day<br>Day 1 vs. 2:<br>estimate = 2.50<br>SE = 1.42<br>df = 86<br>t ratio = 1.758<br>p = 0.3005<br>Day 1 vs. 3:<br>estimate = 3.79<br>SE = 1.43<br>df = 86.1<br>t ratio = 2.651<br>p = 0.0462<br>Day 1 vs. 4:<br>estimate = 5.49<br>SE = 1.42<br>df = 86<br>t ratio = 3.856<br>p = 0.0012<br>Day 2 vs. 4:<br>estimate = 2.99<br>SE = 1.42<br>df = 86<br>t ratio = 2.098<br>p = 0.1621	N/A	No	3B
	Cerebellar-dependent associative motor learning	Preperturbation (step time before obstacle days 5-8)	WT: n = 18 (6F, 12M);<br>dp/+: n = 15 (5F, 10M)	3.3-3.5 mo	Type III ANOVA Satterwaite's Method	F(1,28) = 0.5378<br>SS = 494.0<br>MS = 493.8<br>p = 0.4694	F(1,28) = 0.0417<br>SS = 38.0<br>MS = 38.3<br>p = 0.8396	F(3,84) = 32.7951<br>SS = 90332.0<br>MS = 30110.5<br>p < 0.0001<br>Decreased by day<br>Day 5 vs. 6:<br>estimate = 60.71<br>SE = 8<br>df = 84<br>t ratio = 7.590<br>p < 0.0001<br>Day 5 vs. 7:<br>estimate = 69.35<br>SE = 8<br>df = 84<br>t ratio = 8.670<br>p < 0.0001<br>Day 5 vs. 8:<br>estimate = 63.05<br>SE = 8<br>df = 84<br>t ratio = 7.882<br>p 0.0001<br>Day 6 vs. 8:<br>estimate = 2.34<br>SE = 8<br>df = 84<br>t ratio = 0.293<br>p = 0.9912	N/A	Genotype x Sex<br>F(1,28) = 8.4867<br>SS = 7792<br>MS = 7792<br>p = 0.0070<br>dp/+ F faster than dp/+ M and WT F<br>Female - WT vs. dp/+:<br>estimate = 52.4<br>SE = 23.3<br>df = 28<br>t ratio = 2.248<br>p = 0.0326<br>Male - WT vs. dp/+:<br>estimate = -31.3<br>SE = 16.8<br>df = 28<br>t ratio = -1.863<br>p = 0.0730<br>WT - M vs. F:<br>estimate = 39.0<br>SE = 19.5<br>df = 28<br>t ratio = 1.993<br>p = 0.0561<br>dp/+ - M vs. F:<br>estimate = -44.8<br>SE = 21.1<br>df = 28<br>t ratio = -2.125<br>p = 0.0425	3C
		Postperturbation (step time after obstacle days 5-8)	WT: n = 18 (6F, 12M);<br>dp/+: n = 15 (5F, 10M)	3.3-3.5 mo	Type III ANOVA Satterwaite's Method	F(1,28) = 2.0288<br>SS = 42400<br>MS = 42400<br>p = 0.1654	F(1,28) = 0.0243<br>SS = 507<br>MS = 507<br>p = 0.8773	F(3,84) = 22.0529<br>SS = 1382686<br>MS = 460895<br>p < 0.0001<br>Decreased by day<br>Day 5 vs. 6:<br>estimate = 235.28<br>SE = 38.2<br>df = 84<br>t ratio = 6.165<br>p < 0.0001<br>Day 5 vs. 7:<br>estimate = 275.80<br>SE = 38.2<br>df = 84<br>t ratio = 7.226<br>p < 0.0001<br>Day 5 vs. 8:<br>estimate = 241.82<br>SE = 38.2<br>df = 84<br>t ratio = 6.336<br>p < 0.0001<br>Day 6 vs. 8:<br>estimate = 6.540<br>SE = 38.2<br>df = 84<br>t ratio = 0.171<br>p = 0.9982	N/A	Genotype x Sex<br>F(1,28) = 7.6897<br>SS = 160710<br>MS = 160710<br>p = 0.0098<br>dp/+ F faster than WT F, WT F faster than WT M<br>Female - WT vs. dp/+:<br>estimate = 163.9<br>SE = 63.3<br>df = 28<br>t ratio = 2.588<br>p = 0.0151<br>Male - WT vs. dp/+:<br>estimate = -52.7<br>SE = 45.7<br>df = 28<br>t ratio = -1.152<br>p = 0.2589<br>WT - M vs. F:<br>estimate = 114<br>SE = 53.1<br>df = 28<br>t ratio = 2.155<br>p = 0.0399<br>dp/+ - M vs. F:<br>estimate = -102<br>SE = 57.3<br>df = 28<br>t ratio = -1.784<br>p = 0.0853	3D
Eyeblink Conditioning	CR %	Eyeblink responses	WT: n = 8 (2F, 6M);<br>dp/+: n = 7 (5F, 2M)	4.8-6.8 mo	Linear Mixed Effects Model (Type III), Tukey	F(1, 13) = 0.004856<br>SS = 8.550<br>MS = 8.550<br>p = 0.9455	N/A	F(2.215, 28.79) = 26.09<br>SS = 51678<br>MS = 5739<br>p < 0.0001<br>Increased by session<br>GG ε = 0.2461	N/A	Genotype x Session<br>F(2.215, 28.79) = 3.692<br>p = 0.0335 *<br>GG ε = 0.2461<br><br>Posthoc Tukey:<br>Day 10 - WT vs dp/+<br>Mean diff = 24.68<br>95% CI: 4.125 to 45.23<br>p = 0.0234 *	4A
	CR % (CS only)	Eyeblink responses	WT: n = 8 (2F, 6M);<br>dp/+: n = 7 (5F, 2M)	4.8-6.8 mo	Linear Mixed Effects Model (Type III)	F(1, 13) = 0.3181<br>SS = 8.550<br>MS = 8.550<br>p = 0.5824	N/A	F(3.343, 43.46) = 18.59<br>SS = 51678<br>MS = 5739<br>p < 0.0001<br>Increased by session<br>GG ε = 0.3715	N/A	No	S4A
	CR onset latency	Time to CR onset	WT: n = 8 (2F, 6M);<br>dp/+: n = 7 (5F, 2M)	4.8-6.8 mo	Linear Mixed Effects Model (Type III)	F(1, 13) = 5.024<br>SS = 51914<br>MS = 51914<br>p = 0.0431 *<br>dp/+ > WTs<br><br>WT mean: 212.8<br>16p11.2 Dup mean: 250.1<br>Diff: -37.29<br>95% CI: -73.23 to -1.349	N/A	F(1.987, 25.83) = 2.508<br>SS = 19265<br>MS = 2140<br>p = 0.1014<br>GG ε = 0.2208	N/A	No	4B
	CR onset latency (CS only)	Time to CR onset	WT: n = 8 (2F, 6M);<br>dp/+: n = 7 (5F, 2M)	4.8-6.8 mo	Linear Mixed Effects Model (Type III)	F(1, 13) = 1.154<br>SS = 51914<br>MS = 51914<br>p = 0.3023	N/A	F(3.769, 42.30) = 3.668<br>SS = 19265<br>MS = 2140<br>p = 0.0132 *<br>Increased by session<br>GG ε = 0.4188	N/A	No	S4B
	CR amplitude	Percent eyelid closure	WT: n = 8 (2F, 6M);<br>dp/+: n = 7 (5F, 2M)	4.8-6.8 mo	Linear Mixed Effects Model (Type III)	F(1, 13) = 0.7621<br>SS = 4.243<br>MS = 0.0161<br>p = 0.3985	N/A	F(2.924, 38.02) = 5.594<br>SS = 1.057<br>MS = 0.1175<br>p = 0.0030 **<br>Increased by session<br>GG ε = 0.3249	N/A	No	S4C
	CR amplitude (CS only)	Percent eyelid closure	WT: n = 8 (2F, 6M);<br>dp/+: n = 7 (5F, 2M)	4.8-6.8 mo	Linear Mixed Effects Model (Type III)	F(1, 13) = 0.2612<br>SS = 4.243<br>MS = 0.0161<br>p = 0.6179	N/A	F(3.175, 35.63) = 1.803<br>SS = 1.057<br>MS = 0.1175<br>p = 0.1616<br>GG ε = 0.3528	N/A	No	S4D
Activity Monitoring	Horizontal beam breaks	Hour-by-hour (light)	WT: n = 16 (8F, 8M);<br>dp/+: n = 19 (11F, 8M)	3.0-3.5 mo	GLM	F(1,31) = 3.747<br>SS = 2376762<br>MS = 2376762<br>p = 0.062<br>partial eta squared = 0.108	F(1,31) = 1.228<br>SS = 778713<br>MS = 778713<br>p = 0.276<br>partial eta squared = 0.038	F(2.468,76.511) = 3.619<br>Greenhouse-Geisser<br>p = 0.023<br>partial eta squared = 0.105	N/A	No	S5A
	Horizontal beam breaks	Hour-by-hour (dark)	WT: n = 16 (8F, 8M);<br>dp/+: n = 19 (11F, 8M)	3.0-3.5 mo	GLM	F(1,31) = 1.230<br>SS = 6985322<br>MS = 6985322<br>p = 0.276<br>partial eta squared = 0.038	F(1,31) = 1.395<br>SS = 7922410<br>MS = 7922410<br>p = 0.247<br>partial eta squared = 0.043	F(4.095,126.954) = 15.642<br>Greenhouse-Geisser<br>p < 0.001<br>partial eta squared = 0.335	N/A	No	S5A
	Horizontal beam breaks	Light vs. dark	WT: n = 16 (8F, 8M);<br>dp/+: n = 19 (11F, 8M)	3.0-3.5 mo	MANOVA	N/A	N/A	N/A	N/A	Light:<br>WT vs dp/+<br>mean difference = 152.679<br>SE = 72.648<br>p=0.043<br><br>Dark:<br>WT vs dp/+<br>mean difference = 259.758<br>SE = 240.410<br>p=0.288	S5B
	Vertical beam breaks	Hour-by-hour (light)	WT: n = 16 (8F, 8M);<br>dp/+: n = 19 (11F, 8M)	3.0-3.5 mo	GLM	F(1,29) = 0.795<br>SS = 903087<br>MS = 903087<br>p = 0.380<br>partial eta squared = 0.027	F(1,29) = 0.263<br>SS = 298451<br>MS = 298451<br>p = 0.612<br>partial eta squared = 0.009	F(3.489,101.172) = 4.470<br>Greenhouse-Geisser<br>p =0.004<br>partial eta squared = 0.134	N/A	Hour x Genotype<br>F(3.489,101.172) = 2.575<br>SS = 1280082<br>MS = 1280082<br>p = 0.049<br>partial eta squared 0.082<br><br>Univariate follow up:<br>Only Hour 10 significantly different<br>F(1,29) = 5.468<br>SS = 1751492<br>MS = 1751492<br>p = 0.026<br>partial eta squared 0.159	S5C
	Vertical beam breaks	Hour-by-hour (dark)	WT: n = 16 (8F, 8M);<br>dp/+: n = 19 (11F, 8M)	3.0-3.5 mo	GLM	F(1,29) = 0.147<br>SS = 632064<br>MS = 632064<br>p = 0.705<br>partial eta squared = 0.005	F(1,29) = 0.687<br>SS = 2962931<br>MS = 2962931<br>p = 0.414<br>partial eta squared = 0.023	F(2.309,66.966) = 14.155<br>Greenhouse-Geisser<br>p < 0.001<br>partial eta squared = 0.328	N/A	No	S5C
	Vertical beam breaks	Light vs. dark	WT: n = 16 (8F, 8M);<br>dp/+: n = 19 (11F, 8M)	3.0-3.5 mo	MANOVA	N/A	N/A	N/A	N/A	Light:<br>WT vs dp/+<br>mean difference = -100.420<br>SE = 108.510<br>p=0.362<br><br>Dark:<br>WT vs dp/+<br>mean difference = -78.219<br>SE = 211.836<br>p=0.715	S3D
PPI	PPI %	PPI % = (startle response for pulse alone<br>–<br>startle response for pulse with prepulse<br>/<br>startle response for pulse alone)•100	WT: n = 8 (6F, 4M);<br>dp/+: n = 15 (6F, 4M)	2.7-6.8 mo	Type III ANOVA Satterwaite's Method	F(1,16) = 0.1696<br>SS = 10.43<br>MS = 10.43<br>p = 0.6859	F(1,16) = 1.1119<br>SS = 6.88<br>MS = 6.88<br>p = 0.7423	N/A	F(2,32) = 19.7922<br>SS = 2433.79<br>MS = 1216.90<br>p < 0.0001<br>10 dB vs. 15 dB:<br>estimate = -10.56<br>SE = 2.53<br>df = 32<br>t ratio = -4.172<br>p = 0.0006	Genotype x Sex<br>F(1,16) = 9.3566<br>SS = 575.28<br>MS = 575.28<br>p = 0.0075<br>dp/+ F PPI% < WT F < dp/+ M<br>Female - WT vs. dp/+:<br>estimate = 18.3<br>SE = 6.66<br>df = 16<br>t ratio = 2.744<br>p = 0.0144<br>Male - WT vs. dp/+:<br>estimate= -13.9<br>SE = 8.16<br>df = 16<br>t ratio = -1.709<br>p = 0.1068<br>WT - M vs. F:<br>estimate = 14.4<br>SE = 7.45<br>df = 16<br>t ratio = 1.926<br>p = 0.0720<br>dp/+ - M vs. F:<br>estimate = -17.9<br>SE = 7.45<br>df = 16<br>t ratio = -2.399<br>p = 0.0289<br><br>Decibel x Genotype<br>F(2,32) = 3.2950<br>SS = 405.17<br>MS = 202.59<br>p = 0.0500<br>From 5dB to 10 dB, dp/+ do not significantly change PPI%<br>WT - 10dB vs. 15 dB:<br>estimate = -11.4423<br>SE = 3.58<br>df = 32<br>t ratio = -3.197<br>p = 0.0085<br>WT - 10 dB vs. 5 dB:<br>estimate = 10.1737<br>SE = 3.58<br>df = 32<br>t ratio = 2.843<br>p = 0.0205<br>dp/+ - 10 dB vs. 15 dB:<br>estimate = -9.6756<br>SE = 3.58<br>df = 32<br>t ratio = -2.703<br>p = 0.0286<br>dp/+ - 10 dB vs. 5 dB:<br>estimate = -0.0908<br>SE = 3.58<br>df = 32<br>t ratio = -0.025<br>p = 0.9996	5A
	Startle response	Startle response for pulse alone	WT: n = 8 (6F, 4M);<br>dp/+: n = 15 (6F, 4M)	2.7-6.8 mo	Type III ANOVA Satterwaite's Method	F(1,16) = 0.5108<br>SS = 112433<br>MS = 112433<br>p = 0.4811	F(1,16) = 12.6468<br>SS = 2783909<br>MS = 2783909<br>estimate = -1248<br>SE = 351<br>df = 16<br>t ratio = -3.556<br>p = 0.0026<br>M > F	F(2,32) = 15.2470<br>SS = 6712560<br>MS = 3356280<br>p < 0.0001<br>Reduced by block<br>Block 1 vs. 2:<br>estimate = 407<br>SE = 151<br>df = 32<br>t ratio = 2.691<br>p = 0.0294<br>Block 1 vs. 3:<br>estimate = 836<br>SE = 151<br>df = 32<br>t ratio = 5.522<br>p < 0.0001<br>Block 2 vs. 3:<br>estimate = 429<br>SE = 151<br>df = 32<br>t ratio = 2.831<br>p = 0.0211	N/A	No	5B

Supplementary Table 2. Statistical results for behavior experiments. Significant differences are noted in light orange boxes with bold text.
